# Self-reliance crowds out group cooperation and increases wealth inequality

**DOI:** 10.1038/s41467-020-18896-6

**Published:** 2020-10-14

**Authors:** Jörg Gross, Sonja Veistola, Carsten K. W. De Dreu, Eric Van Dijk

**Affiliations:** 1 Institute of Psychology, Leiden University, Leiden, Netherlands; 2 Center for Research in Experimental Economics and Political Decision Making (CREED), University of Amsterdam, Amsterdam, Netherlands

**Keywords:** Human behaviour, Decision making, Economics, Sociology

## Abstract

Humans establish public goods to provide for shared needs like safety or healthcare. Yet, public goods rely on cooperation which can break down because of free-riding incentives. Previous research extensively investigated how groups solve this free-rider problem but ignored another challenge to public goods provision. Namely, some individuals do not need public goods to solve the problems they share with others. We investigate how such self-reliance influences cooperation by confronting groups in a laboratory experiment with a safety problem that could be solved either cooperatively or individually. We show that self-reliance leads to a decline in cooperation. Moreover, asymmetries in self-reliance undermine social welfare and increase wealth inequality between group members. Less dependent group members often choose to solve the shared problem individually, while more dependent members frequently fail to solve the problem, leaving them increasingly poor. While self-reliance circumvents the free-rider problem, it complicates the governing of the commons.

## Abstract (web summary)

Cooperation among humans is threatened by the free-rider problem. Here the authors identify another challenge to human cooperation: self-reliance, the ability to solve shared problems individually. The experiment reveals that self-reliance crowds out cooperation and increases wealth inequality.

## Introduction

Humans have the ability to establish public goods through cooperation^1–4^. In groups, we can protect ourselves against outside danger, disseminate knowledge and care for the elderly or the sick. The provision of public goods, like public healthcare and public infrastructure, illustrates how cooperation allows humans to achieve more collectively than they could alone^5,6^. The problem is that public goods also introduce a social dilemma: public goods rely on the willingness of each individual group member to contribute to their provision, while consumption is not restricted to those who contribute^4,7–9^. This feature of non-excludability invites exploitation by free-riding. Without an aligned interest to cooperate, groups run the risk that provision levels will be suboptimal to the point that public goods are not provided at all^3,10,11^.

Previous research extensively investigated mechanisms that can solve this problem of free-riding, like punishment^10,12–14^, partner choice^15–17^, or long-term interactions^18,19^. Free-riding is not, however, the only challenge for human cooperation^20–22^. So far ignored is that cooperation may break down because of self-reliance – having the physical or financial resources to solve shared problems independently of groups and group cooperation. For example, shared problems like healthcare, transportation and security can be solved through public goods provision but also, at least by some people, through private means. While self-reliance avoids the free-rider problem altogether, it introduces a different social dilemma: the dilemma between solving problems as a group versus individually. Previous research suggests that the availability of local (excludable) group goods can reduce the provision of global (non-excludable) public goods^23–25^ but leaves open whether this is due to increased free-riding or a preference for self-reliance^22^. Self-reliance is different from free-riding, since individuals solving a problem privately do not benefit from others’ efforts to solve the problem cooperatively. In addition, it is unknown how groups solve shared problems when there is a division between those who can afford to be self-reliant and those who depend on public goods solutions. Previous research has investigated asymmetries in public goods provision problems, for example, by manipulating the resource distribution within groups or the productivity of different group members^21,26–31^ revealing some, albeit mixed^32–35^, evidence that asymmetry can reduce cooperation. Yet, how asymmetries in the ability to become independent of groups influence public goods provision is unknown. Here we confront groups with the dilemma of self-reliance and show that the ability to be self-reliant reduces the efficient provision of public goods. Especially when group members differ in their ability to take care of themselves, we find that self-reliance amplifies wealth inequality and undermines support for community-based solutions.

For our analysis we extend previous public goods provision and coordination problems^11,22,29,36–43^ and provide a general framework to study cooperation when some group members depend on public goods while others can be self-reliant. To illustrate, imagine a group of people who live at the coast and have to prepare for rising sea levels. One option is to build a dam around the entire village. If finished in time, it protects all group members from the dangers of a flood. This option requires cooperation – no single group member alone can build the dam. Yet, imagine a second option which is to build a dam around one’s own house rather than the whole village. If the homeowner has access to enough resources, she can protect herself without having to rely on the group and risk exploitation by free-riders.

**Fig. 1:**
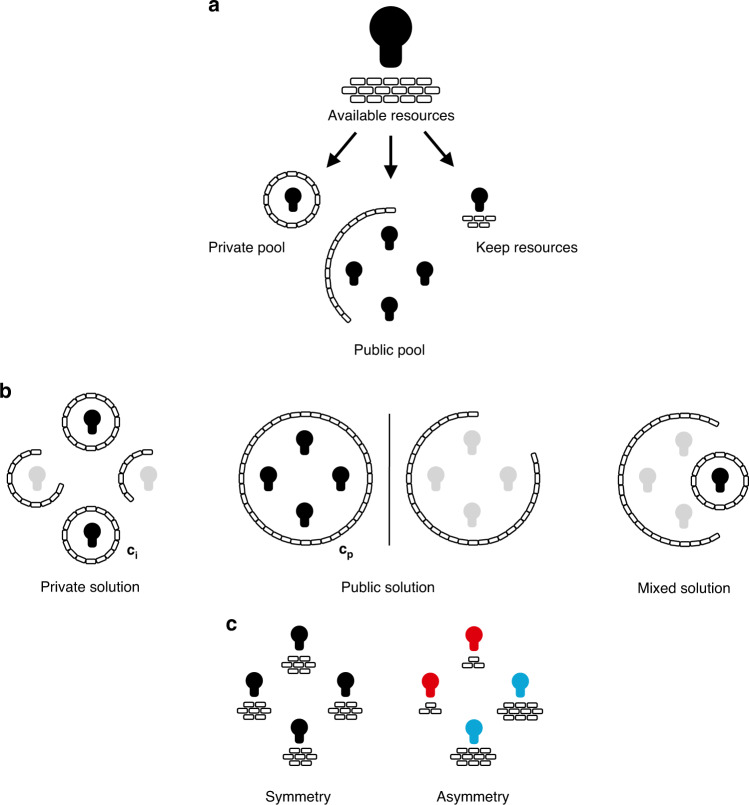
The social dilemma of self-reliance. **a** A group (*n* = 4) is faced with a shared problem. Each group member has to individually decide how many resources she wants to invest into a private pool or into a public pool, keeping the remaining resources for herself. Each group member has to either reach a private solution for a cost *c*_*i*_ or a public solution for a cost of *c*_*p*_ to avoid losing the resources they kept for themselves (with *c*_*i*_ < *c*_*p*_ and *c*_*i*_ ≥ *c*_*p*_/*n*). **b** The private solution only protects the respective group member. The public solution, instead, constitutes a public good. If group members together invest enough resources into the public pool and reach the threshold *c*_*p*_, all group members are protected. If neither threshold *c*_*i*_ nor *c*_*p*_ is reached, the group member loses her remaining units (indicated in grey). **c** In the symmetry condition (*n* = 25 groups), all group members are equally able to solve the problem individually. In the asymmetry condition (*n* = 25 groups), two group members are rather dependent on cooperation (red, low resource availability), while it is easier to be self-reliant for the other two group members (blue, high resource availability).

In our experiments we confronted participants with a stylised version of this collective action problem (Fig. 1, see also the Supplementary Discussion for a more in-depth theoretical motivation and game theoretic analysis). Four group members were assigned to one group. In each round, group members had to simultaneously decide how to allocate their resources towards solving a shared problem through a public or a private solution (Fig. 1a). Each group member needed to solve the problem through either means otherwise they would lose the resources that they did not invest. On average, group members had 90 resource units at their disposal. We set the threshold to create a public solution at *c*_*p*_ = 180 units. Hence, if each group member invests, for example, 45 units, the public good is created and the problem is solved collectively. The public good is non-excludable: if created, the problem is solved for all group members (Fig. 1b). This also means that it is preferable for any single group member if others pay the lion’s share of the cost, as this allows them to free-ride on the cooperation of others – the classic dilemma of cooperation with the inherent risk that the group fails to create a public solution and everybody loses.

In the experiment, the threshold to create a private solution was set at *c*_*i*_ = {∞, 75, 65, 55, 45} across counterbalanced blocks of 10 consecutive rounds (within-group factor with repeated measures). Solving the problem privately constituted a private good that only solves the problem for the respective group member. Under *c*_*i*_ = ∞, private solutions were not attainable for any group member. In this case, solving the problem required cooperation and the problem reduced to a step-level public goods game. When attainable, however, the private solution protects group members from the risk of exploitation and cooperation failure, but also deprives the group from important resources (Fig. 1b). Opting for self-reliance is thus different from free-riding from an economic perspective: a group member that solves the problem privately can no longer benefit from the resources other group members spend on solving the problem cooperatively. Free-riding thus may be motivated by ‘greedy’ attempts to benefit from others’ cooperativeness, but self-reliance cannot. At the same time, both free-riding and opting for self-reliance may be driven by the ‘fear’ that others will exploit one’s own cooperativeness. Self-reliance therefore separates ‘fear’ from ‘greed’ from a psychological perspective.

By varying the cost of the private solution (*c*_*i*_), we manipulated to which degree group members can rely on themselves or depend on group cooperation – by lowering *c*_*i*_ it became easier for group members to simply solve the problem individually. For example, under *c*_*i*_ = 45, group members only had to invest 45 units into their private pool to solve the problem individually. Between conditions, we manipulated the ability of self-reliance within groups, making some group members more dependent on cooperation and public good solutions than others (between-group factor, Fig. 1c). Specifically, in our asymmetry condition, two group members had *e*_high_ = 120 units at their disposal, while the other two group members were endowed with *e*_low_ = 60 units in each round. Hence, regardless of *c*_*i*_, it was always easier for ‘less dependent group members’ (*e*_high_ = 120) to solve the shared problem individually when the private solution was present, while ‘more dependent group members’ (*e*_low_ = 60) more heavily relied on a group solution. In this condition, group members were not only faced with a conflict between cooperation and self-reliance. They also depended on the creation of public goods asymmetrically, similar to unequal access to private healthcare plans, privatised means of protection, or private means of transportation. In our symmetry condition, in contrast, each group member received an equal amount of *e* = 90 units in each round. Thus, every group member was equally able to be self-reliant (or not) across the *c*_*i*_ levels.

## Results

### Decline of collective action

**Fig. 2:**
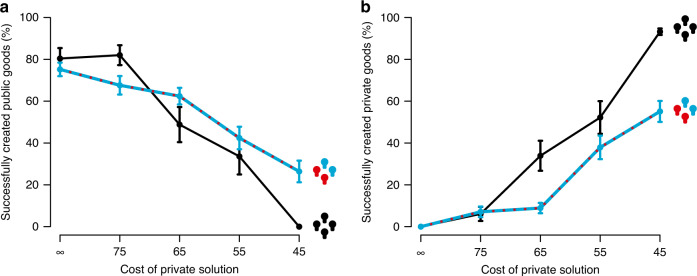
Creation of public and private solutions across declining private solution costs. **a** Successful creation of the public good (cooperation) declined when the cost of the private solution decreased. Cooperation, however, remained more stable when resources were distributed asymmetrically (*n* = 25 groups, blue line) compared to the symmetry condition (*n* = 25 groups) in which group members were equally dependent on each other (black line). **b** Reversely, group members created more private goods and chose to become self-reliant more frequently in the symmetry condition with cheaper private solutions (black line) compared to groups in the asymmetry condition (blue line). Points indicate the mean. Error bars indicate the standard error of the mean.

When cooperation was the only means to avoid losing one’s remaining resources (*c*_i_ = ∞), groups frequently managed to create the public good. On average, 78% of the groups successfully solved the problem through cooperation, regardless of whether resources were distributed equally or unequally (cluster-adjusted *χ*^2^-square test, *P* = 0.27, two-sided, see also refs. ^21,28,30,32,35,37,44,45^ for related findings). Introducing the possibility to become self-reliant led to a steady decline of cooperation. The cheaper the cost of the private solution, the less likely groups solved the shared problem cooperatively across both conditions (Fig. 2a, mixed effects regression, *P* < 0.001, two-sided). When self-reliance was relatively cheap (*c*_*i*_ = 45), none of the groups in which the members were equally dependent on each other reached a public solution and nearly all group members (93%) solved the problem individually (Fig. 2). Interestingly, in the asymmetry condition, 26% of the groups still managed to create a public good and only 55% of the group members solved the problem individually (Fig. 2, aggregated across rounds, symmetry vs. asymmetry condition; Mann–Whitney *U*-test, *U* = 92, *P* < 0.001, two-sided). This indicates that less dependent group members, at least to some degree, spent resources on public solutions even though they did not need to.

### Exploiting the ability of self-reliance

**Fig. 3:**
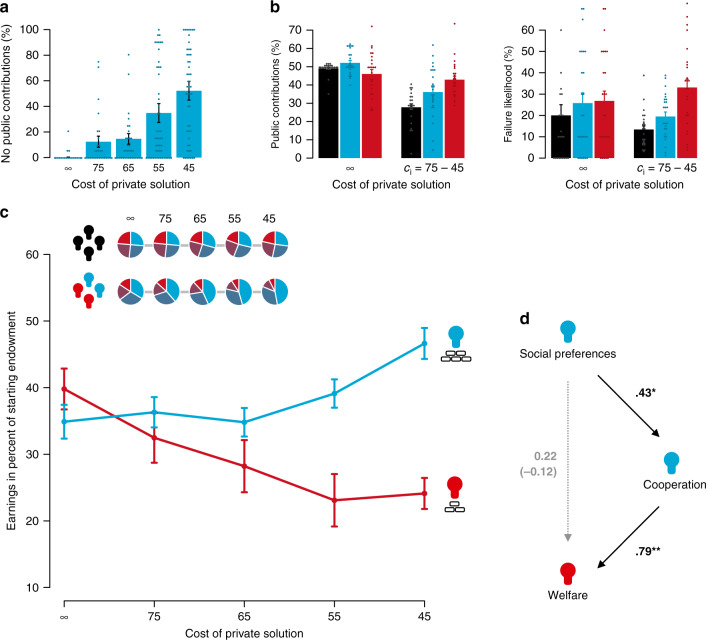
Consequences of self-reliance asymmetry. Less dependent group members (*n* = 50) frequently chose to not contribute anything to the public solution (**a**). In comparison to group members in the symmetry condition (*n* = 100, black/left bars) and less dependent group members (*n* = 50, blue/middle bars), more dependent group members (*n* = 50, red/right bars) dedicated more of their resources to cooperation (left). At the same time, they had the highest risk of losing all of their resources (right) when private solutions could substitute a public solution (**b**). Reducing the cost of private solutions for the group’s shared problem increased earnings disparity between less (blue line) and more dependent (red line) group members. Inset: in comparison to the symmetry condition, wealth inequality increased in the asymmetry condition as private solutions became more attainable (i.e. with lower *c*_*i*_) (**c**). Pro-social preferences of less dependent group members – the degree to which they generally value the welfare of others – predicted contributions to the public solution which, in turn, predicted earnings of more dependent group members (mediation model based on regressions with bootstrapped confidence intervals aggregated across *c*_*i*_ levels, coefficients show standardised path coefficients) (**d**). *P = 0.03, two-sided; **P < 0.001, two-sided. Points indicate individual data points. Bars indicate the mean. Error bars indicate the standard error of the mean.

Although less dependent members may cooperate with more dependent group members because of other-regarding preferences, self-reliance not only decreased overall cooperation but led to a self-enforcing exploitation dynamic when some could afford to be self-reliant whereas others could not. In particular, less dependent group members frequently chose to not contribute anything to the public good (Fig. 3a), thereby ‘forcing’ more dependent group members to compensate for missing resources (see Supplementary Notes 3 and 5 for details). Indeed, when private solutions were available, more dependent group members dedicated most of their resources to cooperation compared to all other group members (Fig. 3b, *e* = 60 vs. 120, comparison within the asymmetry condition, aggregated across rounds, Wilcoxon signed-rank test, *W* = 240, *P* = 0.04, two-sided; *e* = 60 vs. 90, comparison between symmetry and asymmetry condition, aggregated across rounds, Mann–Whitney *U*-test, *U* = 569, *P* < 0.001, two-sided).

Since more dependent group members could not create the public good without at least some support from their less dependent fellow group members, they also had the highest risk of losing all of their remaining resources (Fig. 3b, *e* = 60 vs. 120, aggregated across rounds, Wilcoxon signed-rank test, *W* = 288, *P* < 0.001, two-sided; *e* = 60 vs. 90, aggregated across rounds, Mann–Whitney *U*-test, *U* = 506, *P* < 0.001, two-sided). We also find that less dependent group members used self-reliance strategically, by reverting to individual solutions and withdrawing support from the public solution when cooperation rates of more dependent group members were lower. Especially when private solutions were exclusively available for less dependent group members (*c*_*i*_ = {75, 65}), such withdrawals led to increased cooperation by more dependent group members in response, suggesting that private solutions were also used as a ‘coercion device’ by less dependent group members (see Supplementary Note 5).

### Wealth gap

The group dynamics that emerged due to asymmetric access to private solutions and ability to rely on oneself increased rather than closed the wealth gap between the less and more dependent group members (Fig. 3c). Group members that relied on the creation of public goods the most were left with less and less of their initial resources across the *c*_*i*_ parameter space, leading to a rise in inequality (mixed effects regression, *P* = 0.01, two-sided). In comparison, wealth distribution remained rather stable and uniform when group members were equally dependent on each other (Fig. 3c inset, mixed effects regression, *P* < 0.001, two-sided). When group members had equal access to private solutions, groups were also wealthier compared to groups with an asymmetry in self-reliance (aggregated to the group-level, two-sample *t*-test, *t*(41) = 2.06, *P* = 0.04, two-sided). Across rounds, groups in the symmetry condition increasingly solved the problem cooperatively when private solutions were unavailable (*c*_*i*_ = ∞) or rather expensive (*c*_*i*_ = {75, 65}), indicating that they learned to coordinate on the more efficient communal solution over time. In the asymmetry condition, this was only the case for *c*_*i*_ = ∞ (Supplementary Fig. 15). In this condition, we did not find a significant trend over rounds when private solutions were available, indicating that unequal access to private solutions also diminishes the ability of groups to coordinate on a more efficient communal solution over time (see Supplementary Note 6 for details).

### Social preferences

The decision of less dependent group members to solve the problem individually was largely driven by social preferences. Social preferences were measured in a separate task, the incentivized social value orientation slider measure^46^, in which participants decide whether to allocate money self-servingly or pro-socially between themselves and an unknown other person. The less the participants valued the welfare of others, the less they contributed to the public solution (Fig. 3d, causal mediation model). This means that the welfare of more dependent group members hinged on the luck of having pro-socially inclined less dependent group members in their group.

### Third-party delegation

Given the adverse effects of self-reliance for social welfare and distribution of wealth, we wanted to know whether groups would be willing to delegate their problem to a third party. Whereas a third-party institution that enacts decisions on behalf of others can counteract negative consequences of (unregulated) group decisions^47,48^, the coordination, emergence and legitimacy of a third party depends on the support of the group^13,49,50^. Additional participants (*n* = 61) were invited separately to take part as a third party and were told that they made decisions on behalf of a group. After the last round of each block, group members in both conditions of the main experiment were asked to vote whether they wanted to let another participant (the third party) make a decision on their behalf. If a majority voted for delegation, the decision of the third party was implemented, replacing the last round of the previous block. This way, we could investigate whether (i) third parties would favour more communal solutions, (ii) invest resources in a way that would be more beneficial for more dependent group members and (iii) if group members would anticipate this and reveal self-serving voting preferences.

**Fig. 4:**
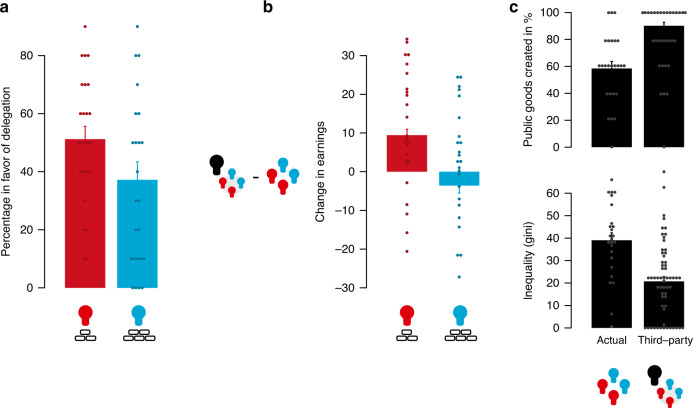
Delegating group decisions to a third party. When asked, a majority of more dependent group members (*n* = 50) voted in favour of delegating the group’s decision to a third party, while only a minority of less dependent group members (*n* = 50) were in favour of delegation (**a**). Solutions favoured by third parties (*n* = 61) would have increased the earnings of more dependent group members at the expense of less dependent group members (**b**). Third parties also more often chose to solve the groups’ problem cooperatively (upper panel) and used the public good to redistribute resources in order to reduce inequality between the more dependent and less dependent group members (lower panel) (**c**). Points indicate individual data points. Bars indicate the mean. Error bars indicate the standard error of the mean.

Figure 4a shows the average voting pattern in the asymmetry condition (see Supplementary Note 4 on results of the symmetry condition). A majority of more dependent group members (51%) were in favour of delegating the group decision to the third party. In contrast, only a minority of the less dependent members (37%) voted in favour of delegating the decision to the third party (aggregated to the endowment-level, paired *t*-test, *t*(24) = 2.20, *P* = 0.04, two-sided). More generally, the more a group member earned in total, the lower the likelihood that the group member voted in favour of delegation (Spearman *r* = −0.41, *P* = 0.003, two-sided). Only 30% of the times, groups successfully installed a third-party decision maker. Compared to the outcome of the actual group interactions, third parties would have lowered the wealth gap between less dependent and more dependent group members substantially (aggregated to the group-level, two-sample *t*-test, *t*(67) = −4.48, *P* < 0.001, two-sided), favouring the more dependent group members at the expense of the less dependent (Fig. 4b). Third parties also favoured group solutions over private solutions (Fig. 4c). From this perspective, the voting pattern of less dependent and more dependent group members reflects their own self-interest.

## Discussion

Especially in modern societies, privatised healthcare, security (like gated communities and private protection), or access to private vs. public transportation influence the interdependence structure of groups and introduce asymmetries in the ability to be self-reliant^51–54^. Self-reliance allows individuals to solve problems independent of groups, creates more freedom of choice and possibly mitigates coercion or groupthink associated with high group-interdependence^55–57^. But, as shown here, it can also create a social dilemma. In this social dilemma of self-reliance, group members that have the ability to be self-reliant may opt for private solutions which inhibits the efficient creation of public goods that benefit all. Indeed, when individuals could solve their (shared) problem privately, overall levels of cooperation reduced, a pattern that resonates with work showing that group members favour local (group excludable) goods over global (non-excludable) public goods^23,24^, and prioritise their own or group interest over concerns for universal welfare^58–64^.

Furthermore, our results point to self-reliance and within-group differences therein to influence redistribution and voting preferences. Policy makers need to be aware that introducing private solutions to shared problems creates a division between those who can afford to be self-reliant and those who heavily depend on cooperation and public goods creation^54,65,66^. On the one hand, when we decreased the endowment of two group members to the point where self-reliance became very costly or impossible, public goods creation increased slightly. Arguably, these group members were forced into higher levels of cooperation due to the lack of alternatives and pro-socially inclined less dependent group members were willing to find a public solution with them. On the other hand, making private solutions viable only for some also widened rather than closed pre-existing wealth gaps in our experiments, resonating with evidence of increasing inequality in modern societies^67,68^.

Humans are a strongly co-dependent species^69–72^. It has been argued that this co-dependence has co-evolved with the ability to overcome the free-rider problem and solve the evolutionary puzzle of cooperation^73–77^. Our results indeed show that groups are perfectly able to coordinate collective action when they depend on it. Such successful cooperation also allows groups to accumulate wealth and increase social welfare over and beyond what individuals alone are capable of. Yet, wealth can provide the ability to solve shared problems individually, alleviating some, but not all, individuals from the immediate dependency on groups^22,78–81^. Paradoxically, this creates a social dilemma of self-reliance that, as shown here, undermines the very reason why group coordination and cooperation may have emerged in the first place: co-dependency. With increased self-reliance, groups increasingly fail to efficiently create public goods which amplifies wealth inequalities, undermines social cohesion and polarises preferences for governing the commons. To mitigate such problems, people either need to establish institutions that increase the willingness to contribute to communal solutions even when some group members can solve shared problems individually, or curb inequality in self-reliance to equalise individual freedom and the degree of group dependence.

## Methods

### Subjects

A total of 261 participants took part in this study. We obtained informed consent from all participants prior to taking part in the experiment. Participants were free to withdraw from participation at any time. Experiments were approved by the Psychology Research Ethics Board of the University of Leiden (file CEP18-1218/494). In all, 200 of our participants were randomly allocated to groups of four, making up 50 groups in total. Half of the groups were assigned to the symmetry condition in which resources were distributed equally (25 groups). The other half of the groups were assigned to the asymmetry condition facing our collective action problem with different resource endowments, thereby creating an asymmetry in the degree to which group members could afford to be self-reliant (25 groups). Further, 61 participants were invited individually, acting as third parties for our groups.

### The private-public goods game

Groups were faced with a dilemma of solving a shared problem either privately or together as a group. Specifically, in each round, group members were individually endowed with resource points (RP) that they had to simultaneously distribute across their own ‘private pool’ or a group-shared ‘public pool’, keeping any RP not invested. After each round, participants would earn the RP that they kept for themselves only if enough RP were invested into either their individual private pool or the shared public pool. If they did not meet either, the threshold *c*_*i*_ of their private pool or the threshold *c*_*p*_ of the shared public pool, they lost all remaining RP that they kept for themselves and earned 0 for that round. Meeting the individual private threshold *c*_*i*_ would only solve the problem for the respective group member, while meeting the public threshold *c*_*p*_ would solve the problem for all group members. The public pool, hence, constitutes a step-level public good that everyone can enjoy if it is created but also carries the risk of free-riding and under-provision. The private pool constitutes a private good. When it is created, it only solves the problem for the respective group member. Note that this also means that self-reliance is different from free-riding from an economic perspective: while group members can free-ride on fellow group members by investing less towards the creation of the public pool, group members who decide to meet their private threshold and solve the problem individually cannot benefit from the cooperation of other group members anymore. The cost to create a public solution was fixed at *c*_*p*_ = 180 RP. That means that, if every group member invested, for example, 45 RP each into the public pool, the public good was created and all group members would keep their remaining RP that they decided to keep for themselves. The cost to create a private solution was varied between blocks of 10 rounds (within-group factor), each taking a value from the set {**∞**, 75, 65, 55, 45}. The block order was counterbalanced across groups. When *c*_*i*_ = **∞**, this meant that the private solution was unattainable for any group member, forcing the group to coordinate on a public solution.

### General procedure

Participants completed the experiment in individual cubicles in front of a computer. After signing informed consent, instructions explained the game to participants, followed by comprehension questions to make sure that every participant understood the rules of the game (see Supplementary Methods for details). The cost-structure was announced before every block alongside the endowment of each group member. Then participants simultaneously decided to assign their resources, followed by a feedback stage that showed (1) how many RP each group member assigned to their private and the shared public pool, (2) which group member(s) met their private target *c*_*i*_, (3) whether the public target *c*_*p*_ was met and (4) how many RP each group member earned in this round.

After the last round of every block, each group member was asked to vote for or against delegating the last round to a third party that would decide the contributions of all four group members. If a majority voted in favour of delegating, the individual decisions of the last round were replaced with the decision from the third party (see also below). Note that group members were not informed about the outcome of the vote or how the third party decided until the very end of the experiment to avoid that participants learned about the voting preferences of fellow group members or the redistribution preferences of third parties over time, which could influence their voting decisions across blocks.

After the main experiment, participants provided fairness evaluations, demographic information and filled out an incentivized lottery task measuring risk-preferences^82^ and the incentivized social value orientation slider measure^46^. In this measure of social preferences, participants decide how to allocate points between themselves and an unknown other person. Points could be allocated self-servingly or pro-socially (sacrificing points to benefit the other person), allowing us to estimate a participant’s social preferences. Participants were paid for three randomly selected rounds from every *c*_*i*_ block. In total, 100 RP were worth 0.50€ paid in cash after the experiment, on top of a fixed show-up fee of 6.50€ and earnings from the social value orientation slider and risk measure. The experiment took ~50–60 min and participants earned, on average, 11.60€.

### Self-reliance manipulation

Across groups, we manipulated the resource distribution between group members (between-group factor). In the asymmetry condition, two group members were endowed with 60 RP, while the other two group members were endowed with 120 RP in every round, creating a situation in which some group members depended more on the public solution while others had the ability to ‘take care of themselves’. In the symmetry condition, all four group members started every round with 90 RP and hence were equally dependent on cooperation vs. able to be self-reliant (depending on *c*_*i*_). Note that in both conditions, groups had 360 RP in total at their disposal.

### Experimental design

The main experiment followed a 2 between group (symmetry vs. asymmetry) × 5 within group (*c*_*i*_ = {**∞**, 75, 65, 55, 45}) × 10 repeated measure (10 rounds per within-factor; 50 rounds in total) design. Within the asymmetry condition, participants differed in their endowment (between-subject factor: *e* = 60 vs. *e* = 120). Participants had either a high or a low endowment across the entire experiment to avoid reciprocity or perspective-taking that could emerge if people experience, both, having a low vs. high endowment and switch roles. Data analyses were performed in R (version 3.3.3).

### Third-party condition

Participants assigned to the third-party condition (*n* = 61) were confronted with the same social dilemma, but from a third-party perspective. They were told that they had to make decisions on how to distribute resources for a group of four. Specifically, for each *c*_*i*_ level and resource distribution (*c*_*i*_ = {**∞**, 75, 65, 55, 45} × symmetric vs. asymmetric = 10 decisions in total), they indicated how each individual group member should allocate their resources towards their private or the shared public solution. If a group voted in favour of delegation, a third-party decision was chosen randomly out of all third-party decisions from the respective resource distribution and *c*_*i*_ level and implemented by the computer. Third parties were told that they ‘make decisions on behalf of other groups’ and that ‘some of your decisions may be randomly selected and implemented in other groups that actually engage in this decision-making problem from a ‘first-person’ perspective’. Participants in the third-party condition received a flat show-up fee of 3.50€ for their participation. The experiment took ~20–30 min.

### Reporting summary

Further information on research design is available in the Nature Research Reporting Summary linked to this article.

## Supplementary information

Supplementary Information

Reporting Summary

## Data Availability

All data for the experiments is publicly available in an Open Science Framework (OSF) repository (https://osf.io/twv7b/). Source data are provided with this paper.

## Source data

Source Data

## Code availability

Analysis code for all experiments are also publicly available in the same OSF repository (https://osf.io/twv7b/).
