# Supplementary material for: Self-reliance crowds out group cooperation and increases wealth inequality
Source: Nat Commun. 2020 Oct 14;11:5161. doi: 10.1038/s41467-020-18896-6 (PMC7560835; doi:10.1038/s41467-020-18896-6)
Supplement: Supplementary file 1 — Supplementary Information [file 41467_2020_18896_MOESM1_ESM.pdf]

Supplementary Information

Self-reliance crowds out group cooperation and increases wealth inequality

Gross et al.

Supplementary Discussion

Humans are ill-adapted to care for themselves and rely on groups to satisfy shared needs like food, shelter, or safety[1]. It has been argued that human co-dependence has co-evolved with mental faculties needed to coordinate and sustain cooperation and collective action in groups, ultimately allowing to overcome the cooperation dilemma and solve the evolutionary puzzle of cooperation[2,3]. Arguably, however, changes in ecology and institutions change the interdependence structure of groups and their group members. In this paper, we investigate how groups coordinate collective action problems when group members are able to solve shared problems individually to different degrees. Theoretically and empirically, cooperation depends on reciprocity, the willingness to jointly invest resources for the common good of the group. With the advent of market economies, specialization, and global increase in wealth, however, modern societies provide private solutions to shared problems that enable some but not all of its members to become self-reliant. Private solutions allow the replacement of social interactions, that are based on direct reciprocity, with the exchange of money for goods and services (see also [4-6]). Essentially, resource abundance (either interpreted as ability or wealth) allows one to become self-reliant and reduces the need for direct reciprocity. Real-world examples of such public-private substitution include public transportation that allows many people to travel from A to B but also requires people to contribute (e.g., by buying train tickets and paying taxes) and coordinate (e.g. by being at a certain time at the same place). Access to private transportation by car is an individualized solution to the same problem that avoids co-dependence on others, yet also is energetically more wasteful and can be personally costlier. Further, in modern two-tier healthcare systems, privatized healthcare providers exist next to publicly funded health-care plans. Retirement planning in Western countries increasingly is a mixture of private investment plans and government-regulated public goods provisions[7], and next to publicly funded law enforcement, many citizens acquire home security from private companies or own firearms.

While it can be argued that the increase in wealth and the emergence of market economies reduced the immediate interdependence for all members of society, people also differ in the degree to which they depend on public goods and in the degree to which they have access to the private solutions that substitute joint action. Self-reliance has advantages in the sense that it protects agents from the risk of coordination failure and the risk of being exploited by free-riders, the classic dilemma of cooperation. At the same time, the option to become self-reliant creates a new dilemma that has been largely overlooked in the literature on cooperation: Private

goods introduce a negative social externality. The resources spent on private goods are not spent on public goods provision and hence do not benefit the group as a whole. Here we introduce the 'private-public goods game' that attempts to capture the dilemma of self-reliance and experimentally investigate the trajectory of group cooperation and coordination when groups not only face the dilemma of free-riding and coordination failure, but also the dilemma of self-reliance and its social externality.

Game-theoretic description

The private-public goods game is a variant of a step-level public goods game. It deviates from commonly employed step-level public goods game in two important ways: First, not reaching the threshold leads to losing all remaining resource points (RP) rather than gaining a fixed price (see also [8-11]). Second, group members have an additional strategy to avoid losing RP that only applies to them (private solution).

What follows is a game-theoretic description of the one-shot private-public goods game. There are n players, who are endowed with x_k RP. Each player k simultaneously decides how much of the RP she spends on the public solution $s_{k,p}$, or on the individual, private solution $s_{k,i}$. A strategy of player k is then a pair $(s_{k,p}, s_{k,i})$, with $s_{k,i}, s_{k,p} \geq 0$ and $s_{k,i} + s_{k,p} \leq x_k$. Pairs satisfying these constraints constitute the strategy set S_k of player k. Let c_p be the cost of the public solution and c_i the cost of the private solution. Then, a public solution is realized if $\sum_k s_{k,p} \geq c_p$, whereas player k reaches her private solution if $s_{k,i} \geq c_i$. If a public solution is reached, and/or if player k reaches her private solution, then the payoff of player k is $\pi_k = x_k - s_{k,p} - s_{k,i}$. If neither solution is reached, then the payoff of player k is 0, instead. Resources invested towards the private or public solution, while not reaching the respective target (c_p/c_i) are considered wasted. It follows that any strategy $(s_{k,p} > 0, s_{k,i} > 0)$ is dominated by $\left(s_{k,p} \geq 0, s_{k,i} = 0\right)$ or $\left(s_{k,p} = 0, s_{k,i} \geq 0\right)$. An equilibrium strategy for a rational player would never assign resources to both the individual and public pool, since only one solution needs to be reached.

In our experiments, we set $n = 4$, $c_p = 180$, while c_i was taking values from the set $\{\infty, 75, 65, 55, 45\}$ and RP were either distributed equally ($\mathbf{x} = [90, \dots, 90]$) or unequally ($\mathbf{x} = [60, 60, 120, 120]$) across players. Regardless of the RP distribution, with $c_i > 45$, players choosing their private solutions is Pareto-dominated by all of the public/collective solutions. Further, an equilibrium in which all group members choose the private solution is payoff-

dominated by the equilibrium in which all group members invest 45 RP to the public solution for $c_i > 45$.

Equilibria take the shape $(\bar{s}_{k,p}, 0)_{k\in 1,\ldots,n}$ with $\bar{s}_{k,p} \leq c_i$ (individual rationality) and $\sum_k \bar{s}_{k,p} = c_p$ (collective solution reached without waste). Including the single symmetric solution, the number of pure-strategy equilibria with a public solution for $\mathbf{x} = [90, \ldots, 90]$ is 1 ($c_i = 45$), 12,341 ($c_i = 55$), 89,161 ($c_i = 65$), 237,761 ($c_i = 75$). Including the single symmetric solution, the number of pure-strategy equilibria with a public solution for $\mathbf{x} = [60, 60, 120, 120]$ is 1 ($c_i =$ 45), 12,341 ($c_i = 55$), 61,686 ($c_i = 65$), 118,486 ($c_i = 75$). Note that, except for the single symmetric solution, all pure-strategy equilibria with a public solution entail free-riding: Some group members invest less into creating the public solution than others and hence benefit from the asymmetric cooperation in the group. The private solution cost sets an upper boundary on the extent of free-riding that is possible in equilibrium, since rational agents are not willing to invest more resources towards a public solution than their private alternative. Importantly, self-reliance (reaching the private solution) is different from free-riding from an economic perspective. When an agent opts for self-reliance, she does not benefit from the creation of a public good in any way. However, the psychological motivation to opt for self-reliance can share some similarities to free-riding as discussed in the main manuscript.

The analogy of the private-public goods game with the classic step-level public goods games suggests that there may exist a symmetric mixed-strategy Nash-equilibrium with positive probabilities assigned to contributing to the public solution. In addition, there may be a large number of asymmetric mixed-strategy equilibria[12]. Note that compared to a classic step-level public goods game in which a certain contribution needs to be reached to attain a prize that is equally shared across group members, a situation in which all players choose $(0, 0)$ does not constitute an equilibrium. This is because group members are always better off to reach their private solution as long as $c_i < x_k$.

Supplementary Methods

Participants were invited in groups of four and randomly assigned to the symmetry condition (n = 25 groups; 100 participants) or the asymmetry condition (n = 25 groups; 100 participants). In both conditions, groups were confronted with the 'private-public goods dilemma' across multiple rounds. Conditions differed only with regards to the resource distribution across group members. In the symmetry condition, all group members were endowed with 90 RP (referred to as 'monetary units (MU)' in the experiment) each round. In the asymmetry condition, two group members were endowed with 120 RP each round ('less dependent group members'), while the other two group members were endowed with 60 RP ('more dependent group members') based on random assignment. We further invited 61 participants in the role of third-party decision makers (see below).

Supplementary Figure 1 provides a general overview of the structure of the experiment. Participants received extensive instructions, including examples illustrating the economic game we confronted them with (Supplementary Figure 2). Throughout the experiment we used neutral labels to avoid framing or demand effects. Each group member had to answer a set of comprehension questions (Supplementary Figure 3) before starting the experiment. Two experimenters were available to clarify questions or misunderstandings. Before each block, participants were reminded about how many RP they and each other fellow group member had available, how many RP had to be assigned to the 'public pool' in order to meet the public threshold (c_p) and how many RP had to be assigned to their individual 'private pool' in order to meet the individual/private threshold (c_i) (Supplementary Figure 4).

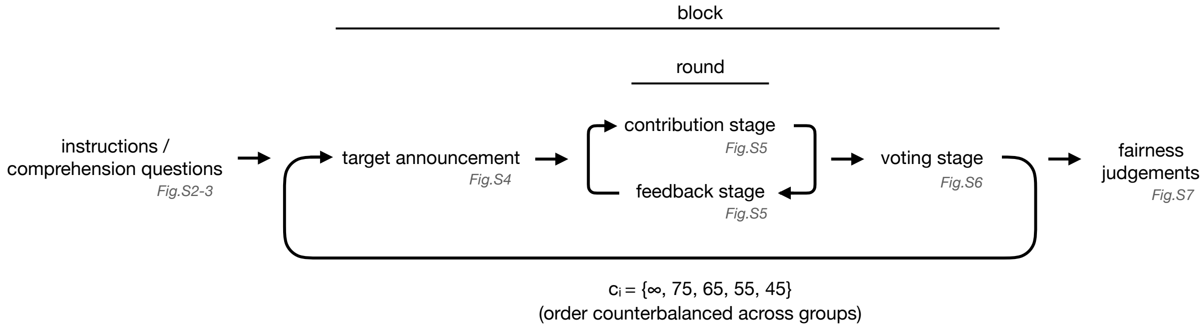

Supplementary Figure 1. Experimental Design. Flowchart illustrating the structure of the experiment.

Across the entire experiment, c_p was fixed to 180 RP. Hence, if every group member would invest, for example, 45 RP into the public pool, the public good was created and all group members would keep any RP that were not spent on the public or private pool. Across five blocks we varied the cost of the private solution between $c_i = \{\infty, 75, 65, 55, 45\}$. The order of these blocks was counterbalanced across groups using the same counterbalance scheme across the symmetry and asymmetry condition. This manipulation allowed us not only to study how groups solved their shared problem when a private solution was available (i.e. $c_i < \infty$) vs. not (i.e. $c_i = \infty$), but also what happens to cooperation and group coordination when the cost of the private solution decreases and, hence, becomes more attainable. For less dependent group members (120 RP) it was always possible to reach a private solution and required relatively less resources compared to more dependent group members (60 RP). In the symmetry condition, it was always possible to reach a private solution and required the same relative amount of resources from each group member. Hence, group members in this condition were equally dependent on public solutions.

Each block consisted of 10 rounds. Each round comprised a contribution stage and a feedback stage (Supplementary Figure 5). In the contribution stage, each group member had to decide how many RP to invest into the shared public pool (s_p), their own private/individual pool (s_i), and how many RP they wanted to keep for themselves. Any RP assigned to the public and private pool was 'spent' and hence did not count towards payment. Instead, group members earned any RP that they did not invest if enough RP were invested into their private pool ($s_i \geq c_i$) *or* enough RP were invested into the shared public pool ($\sum_{k=1}^{4} s_{k,p} \geq c_p$). If neither threshold was reached, the group member lost all remaining RP and earned 0. After all group members made their decision, they saw the round outcome on the feedback screen, showing individual decisions and group outcomes successively to avoid presenting too much information at once. The feedback screen first showed how many RP each group member assigned to the public and private pool. After pressing a button, information was added on how many RP were in the public pool and the group member's private pool in total. Then, it was revealed if the private target was met for each group member and if the group, together, met the public target. Finally, round earnings for each group member were revealed. Participants could then proceed to the next round (Supplementary Figure 5). In one block of the experiment ($c_i = \infty$ block), none of the group members had a private/individual pool available and could only choose how many RP to keep and how many RP to invest into the shared public pool (see bottom panel in Supplementary Figure 4 & 5). In this block, participants were told upfront that

“there is no private pool in this part of the experiment. In this part you and the other group members can only invest resource points into the public pool to reach the public target”.

After the final round of each block and before starting the next block, participants completed the voting stage (Supplementary Figure 6). In this stage, we explained to participants that we invited other people in the role of a third party. These third parties made decisions on behalf of each member in the group. Each group member was then asked to vote in favour or against delegating the last round of the block to this third party. If a majority of the group members ($n > 2$) voted in favour of delegating the last round, the outcome of this round was replaced with the decision of the third party. We chose the last round (instead of all rounds or a random round), because we expected that group members would more frequently mis-coordinate their actions in the first rounds of each block but would converge to a stable outcome by the last round. How other group members voted and the voting outcome was not revealed before the very end of the experiment to avoid that groups learned about the redistribution preferences of third-parties or the voting preferences of fellow group members over time.

After finishing the last block of the private-public goods dilemma, participants moved to the fairness judgment stage (Supplementary Figure 7). In this stage, group members were asked how they would distribute not only their own resources but the resources of all group members, if they would have the ability to do so. Hence, for each c_i level, they were asked to allocate the resources of all group members, themselves included. Note that this decision differed from third-party decisions, since fairness judgments included themselves and, hence, participants could allocate resources self-servingly. We also did not incentivize this part of the task and, instead, emphasized to participants that their decision in this task would not count towards their final payoff. This allowed us to contrast what participants would communicate to us as a fair or desirable distribution of resources with the decisions of impartial third parties and participants’ decisions in the voting stage (that were payoff relevant). Results of the fairness judgment stage are reported in the ‘Supplementary Results’ part below.

The experiment concluded with measuring individual level risk preferences, social preferences, and a demographics questionnaire. Social preferences were measured with the incentivized social value orientation slider measure[13]. In this task, participants have to make multiple decisions on how to allocate points between themselves and an unknown other person. Points can be allocated self-servingly or pro-socially (sacrificing points to benefit the other person) similar to a dictator game. For example, for one item, participants have to choose one of nine possible allocations ranging from allocating 100 points to oneself and 50 points to the other

person (maximal pro-self option) to allocating 50 points to oneself and 100 points to the other person (maximal pro-social option). The slider-measure yields a single score (Social Value Orientation angle; SVO angle) that measures social value orientation as the trade-off between maximizing payoff for oneself and the other person. The higher the SVO angle, the more one is willing to sacrifice points in order to benefit another person (i.e. the degree of other-regarding concerns / social preferences). Risk preferences were measured using the lottery task from the Preference Survey Module[14] that confronts participants with 32 binary choices between choosing a lottery of receiving 300 points with $p = 0.5$ and 0 points with $1-p = 0.5$ or a sure payoff that varies between 0 and 310 points (in steps of 10). 100 points were worth 0.50€.

Third party condition

Shortly before the main experiment, we started to invite 61 participants in the role of third parties. They received similar instructions, explaining the structure and rules of the private-public goods dilemma. However, they did not take part in this dilemma. Instead, we explained to them that they would make decisions on behalf of another group and that their decisions may be implemented and actually affect earnings of real groups. Akin to our main experiment, they made one decision per c_i level, allocating the RP of each individual group member across the public and private pool and deciding how many RP each group member should keep. In total, they made 10 decisions: Five decisions for a group with unequal endowments (for all possible $c_i = \{\infty, 75, 65, 55, 45\}$) and five decisions for groups with equal endowments (for all possible $c_i = \{\infty, 75, 65, 55, 45\}$). Third parties were not affected by how they distributed resources. Hence, they were completely impartial to the outcome and had no incentive to favour one group member over another.

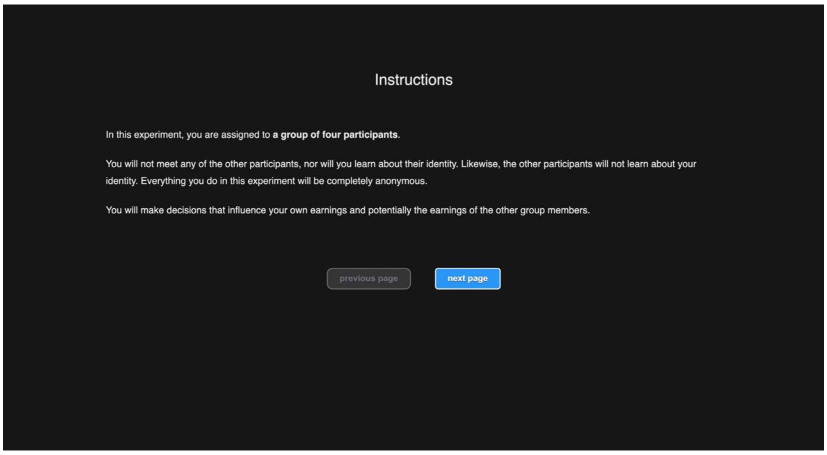

page 1

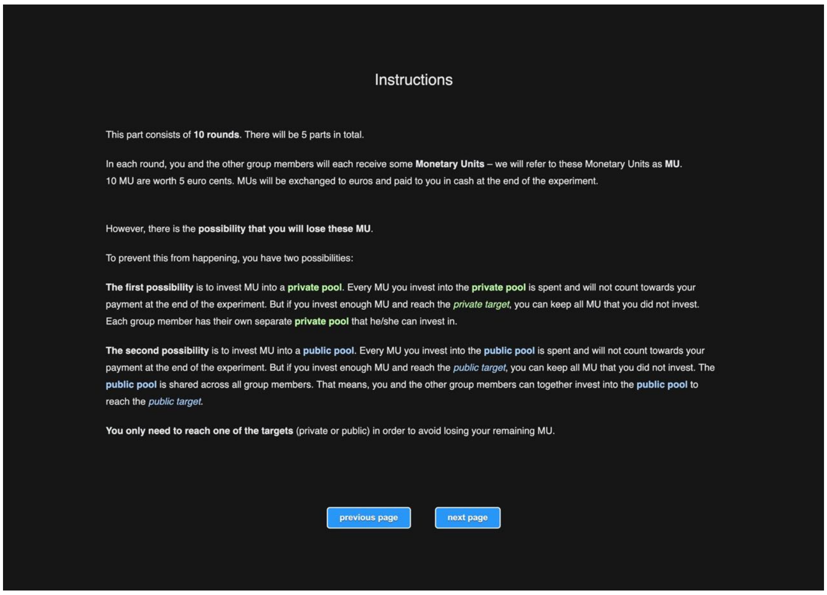

page 2

Supplementary Figure 2. Instructions.

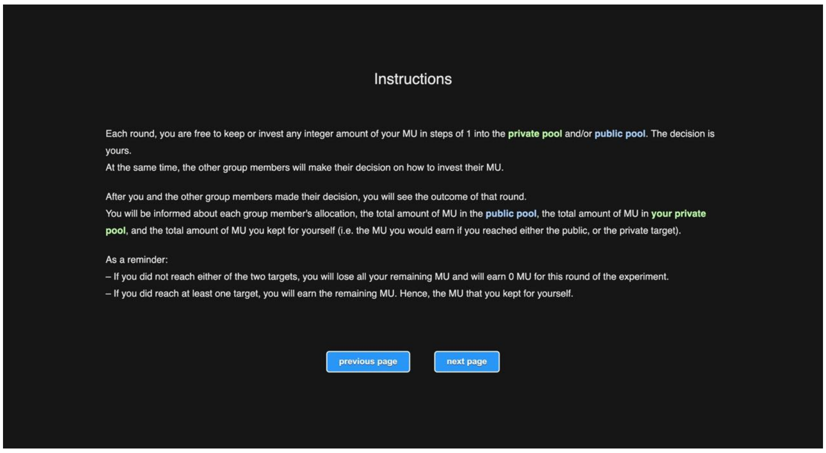

page 3

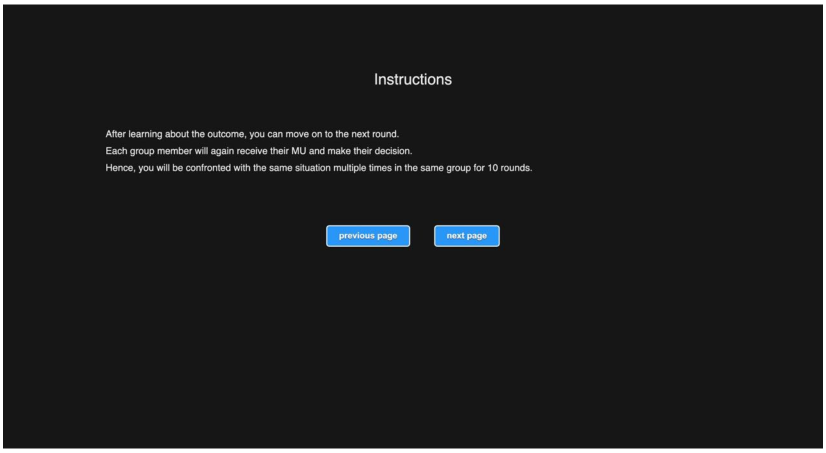

page 4

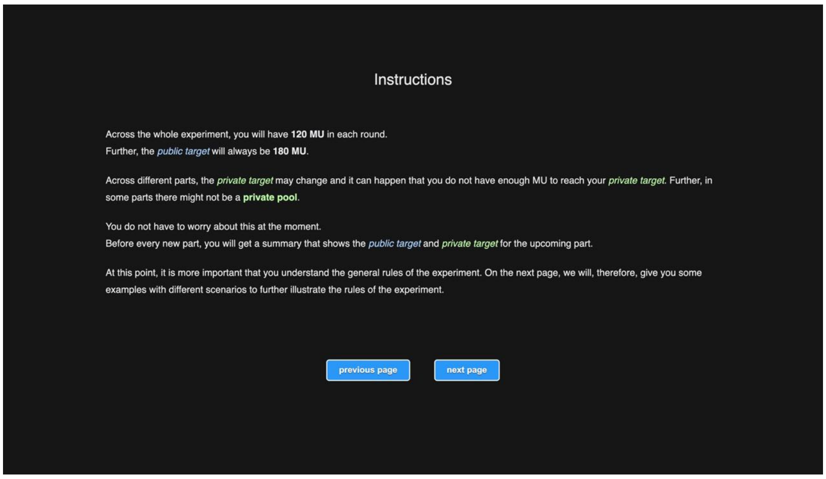

page 5

Supplementary Figure 2. Instructions (continued).

Instructions

The following examples will illustrate the rules of this experiment. These examples are for illustrative purposes only. They do not necessarily have to make sense, but are aimed at illustrating the rules of the task.

Example 1

The *public target* is 180 MU.
The *private target* is 60 MU.

Group member 1 decides to invest **60 MU** into **his/her private pool** and **0 MU** to the **public pool**.
Group member 2 decides to invest **60 MU** into **his/her private pool** and **20 MU** to the **public pool**.
Group member 3 decides to invest **0 MU** into **his/her private pool** and **60 MU** to the **public pool**.
Group member 4 decides to invest **0 MU** into **his/her private pool** and **20 MU** to the **public pool**.

Hence, together, group members 1, 2, 3 and 4 invested **100 MU** into the **public pool** in total.

In this example, group members 3 and 4 lose all their remaining MU, as they, neither, reached their *private target*, nor the *public target*.
Group member 1 can keep his/her remaining MU, because he/she reached *his/her private target*.
Group member 2 can keep his/her remaining MU, because he/she reached *his/her private target*.

Example 2

The *public target* is 180 MU.
The *private target* is 50 MU.

Group member 1 decides to invest **0 MU** into **his/her private pool** and **90 MU** to the **public pool**.
Group member 2 decides to invest **0 MU** into **his/her private pool** and **70 MU** to the **public pool**.
Group member 3 decides to invest **0 MU** into **his/her private pool** and **20 MU** to the **public pool**.
Group member 4 decides to invest **0 MU** into **his/her private pool** and **0 MU** to the **public pool**.

Hence, together, group members 1, 2, 3 and 4 invested **180 MU** into the **public pool** in total.

In this example, none of the group members reached their *private target*.
Still, group members 1-4 can keep his/her remaining MU, because they reached *the public target*.

Example 3

The *public target* is 180 MU.
The *private target* is 70 MU.

Group member 1 decides to invest **0 MU** into **his/her private pool** and **60 MU** to the **public pool**.
Group member 2 decides to invest **0 MU** into **his/her private pool** and **80 MU** to the **public pool**.
Group member 3 decides to invest **0 MU** into **his/her private pool** and **30 MU** to the **public pool**.
Group member 4 decides to invest **70 MU** into **his/her private pool** and **0 MU** to the **public pool**.

Hence, together, group members 1, 2, 3 and 4 invested **170 MU** into the **public pool** in total.

In this example, group members 1, 2, and 3 lose all their remaining MU, as they, neither, reached their *private target*, nor the *public target*.
Group member 4 can keep his/her remaining MU, because he/she reached *his/her private target*.

previous page | next page

page 6 (examples)

Instructions

Payment

As mentioned before, the experiment consists of several parts and rounds.

At the end of the experiment, three rounds from *each* part will be randomly selected for payment.
You will receive the sum of MU you earned in these rounds as euros in cash after the experiment.

Since you do not know which rounds will count for real, you should treat each round independently and as if this round is the one that counts.

Please make sure you fully understand the rules of this experiment. If you have any questions now, please contact the experimenter. Next, we will ask you to answer some comprehension questions to make sure that all participants understand the rules of the experiment before we start with the first part.

Reminder: This study falls under the no deception policy at Leiden University. This means that there are no hidden information and no deception: Everything will happen as stated in the instructions. Furthermore, your decisions and the decisions of the other participants will have real consequences.

previous page | I understood the instructions

page 7 (payment)

Supplementary Figure 2. Instructions (continued).

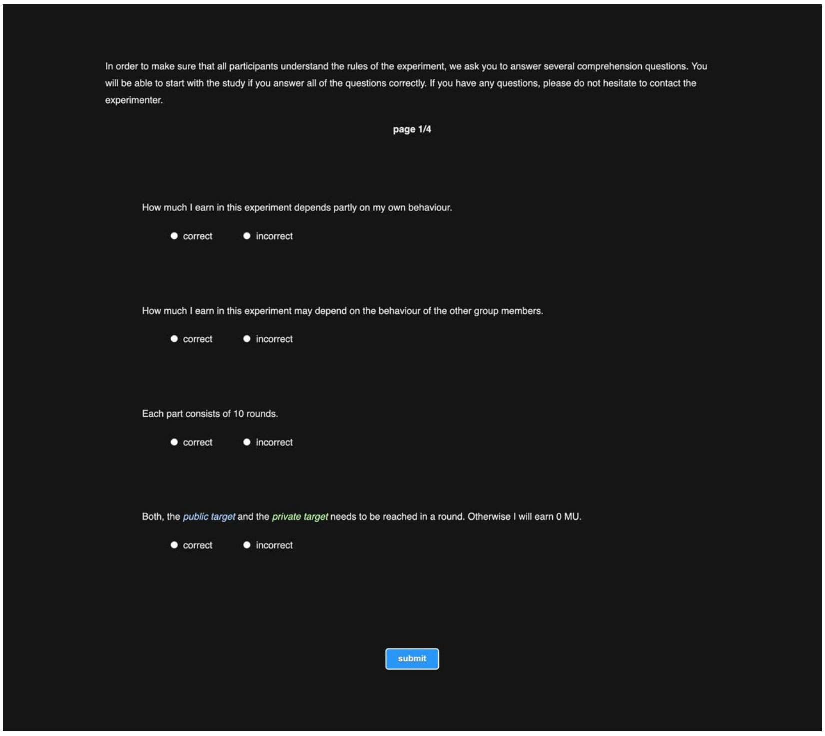

comprehension check 1

Supplementary Figure 3. Comprehension check.

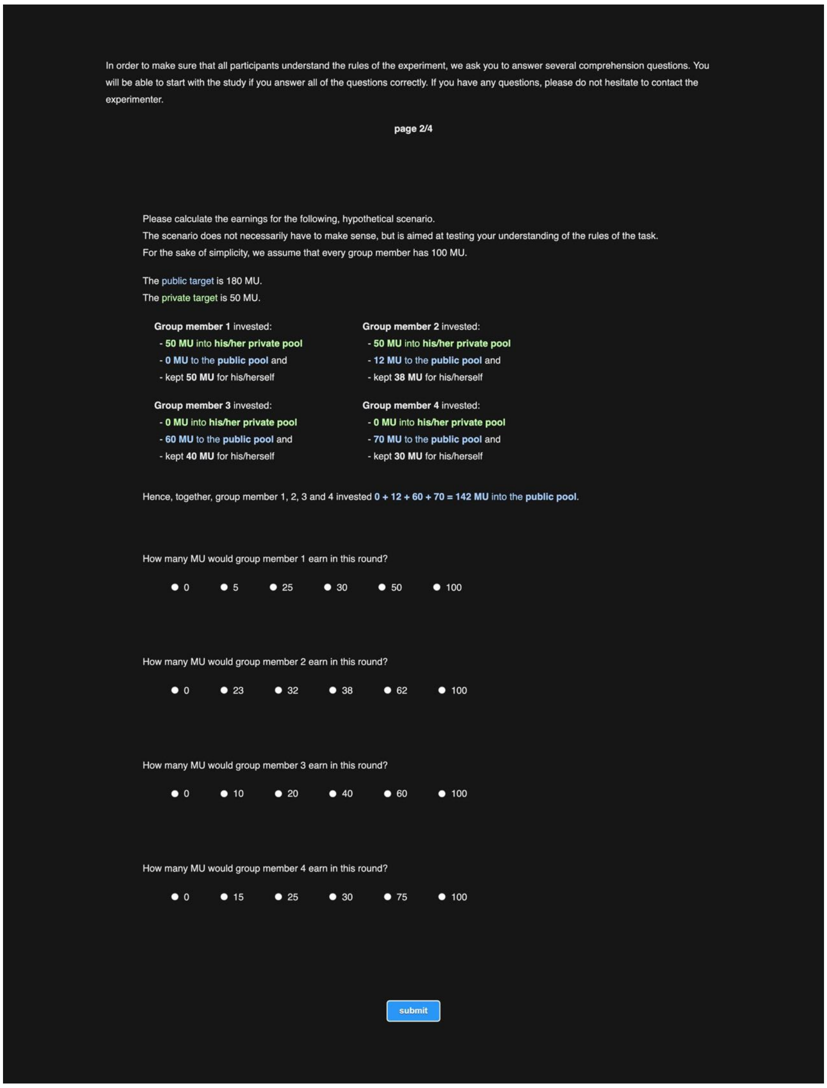
In order to make sure that all participants understand the rules of the experiment, we ask you to answer several comprehension questions. You will be able to start with the study if you answer all of the questions correctly. If you have any questions, please do not hesitate to contact the experimenter.

page 2/4

Please calculate the earnings for the following, hypothetical scenario.
The scenario does not necessarily have to make sense, but is aimed at testing your understanding of the rules of the task.
For the sake of simplicity, we assume that every group member has 100 MU.

The public target is 180 MU.
The private target is 50 MU.

Group member 1 invested:
- **50 MU** into **his/her private pool**
- **0 MU** to the **public pool** and
- kept **50 MU** for his/herself

Group member 2 invested:
- **50 MU** into **his/her private pool**
- **12 MU** to the **public pool** and
- kept **38 MU** for his/herself

Group member 3 invested:
- **0 MU** into **his/her private pool**
- **60 MU** to the **public pool** and
- kept **40 MU** for his/herself

Group member 4 invested:
- **0 MU** into **his/her private pool**
- **70 MU** to the **public pool** and
- kept **30 MU** for his/herself

Hence, together, group member 1, 2, 3 and 4 invested **0 + 12 + 60 + 70 = 142 MU** into the **public pool**.

How many MU would group member 1 earn in this round?

0 5 25 30 50 100

How many MU would group member 2 earn in this round?

0 23 32 38 62 100

How many MU would group member 3 earn in this round?

0 10 20 40 60 100

How many MU would group member 4 earn in this round?

0 15 25 30 75 100

submit

comprehension check 2

Supplementary Figure 3. Comprehension check (continued).

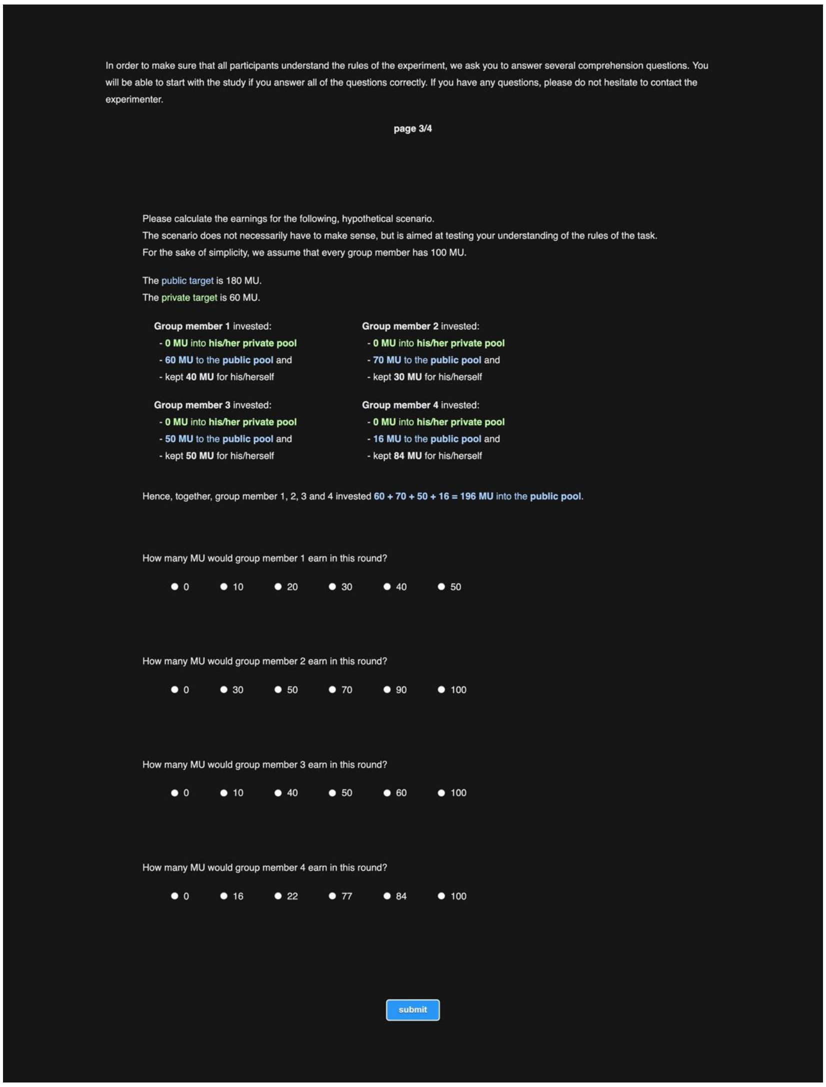

In order to make sure that all participants understand the rules of the experiment, we ask you to answer several comprehension questions. You will be able to start with the study if you answer all of the questions correctly. If you have any questions, please do not hesitate to contact the experimenter.

page 3/4

Please calculate the earnings for the following, hypothetical scenario.
The scenario does not necessarily have to make sense, but is aimed at testing your understanding of the rules of the task.
For the sake of simplicity, we assume that every group member has 100 MU.

The public target is 180 MU.
The private target is 60 MU.

Group member 1 invested:
- **0 MU** into **his/her private pool**
- **60 MU** to the **public pool** and
- kept **40 MU** for his/herself

Group member 2 invested:
- **0 MU** into **his/her private pool**
- **70 MU** to the **public pool** and
- kept **30 MU** for his/herself

Group member 3 invested:
- **0 MU** into **his/her private pool**
- **50 MU** to the **public pool** and
- kept **50 MU** for his/herself

Group member 4 invested:
- **0 MU** into **his/her private pool**
- **16 MU** to the **public pool** and
- kept **84 MU** for his/herself

Hence, together, group member 1, 2, 3 and 4 invested **60 + 70 + 50 + 16 = 196 MU** into the **public pool**.

How many MU would group member 1 earn in this round?

● 0 ● 10 ● 20 ● 30 ● 40 ● 50

How many MU would group member 2 earn in this round?

● 0 ● 30 ● 50 ● 70 ● 90 ● 100

How many MU would group member 3 earn in this round?

● 0 ● 10 ● 40 ● 50 ● 60 ● 100

How many MU would group member 4 earn in this round?

● 0 ● 16 ● 22 ● 77 ● 84 ● 100

submit

comprehension check 3

Supplementary Figure 3. Comprehension check (continued).

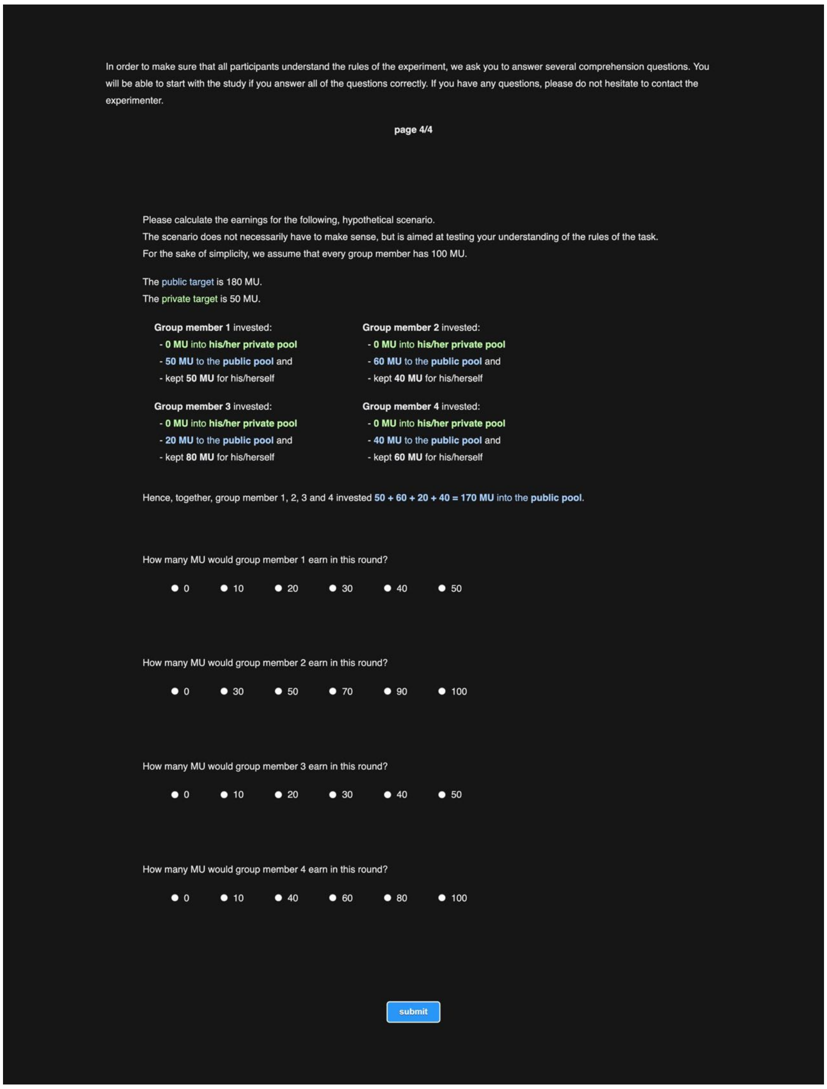
In order to make sure that all participants understand the rules of the experiment, we ask you to answer several comprehension questions. You will be able to start with the study if you answer all of the questions correctly. If you have any questions, please do not hesitate to contact the experimenter.

page 4/4

Please calculate the earnings for the following, hypothetical scenario.
The scenario does not necessarily have to make sense, but is aimed at testing your understanding of the rules of the task.
For the sake of simplicity, we assume that every group member has 100 MU.

The public target is 180 MU.
The private target is 50 MU.

Group member 1 invested:
- **0 MU** into **his/her private pool**
- **50 MU** to the **public pool** and
- kept **50 MU** for his/herself

Group member 2 invested:
- **0 MU** into **his/her private pool**
- **60 MU** to the **public pool** and
- kept **40 MU** for his/herself

Group member 3 invested:
- **0 MU** into **his/her private pool**
- **20 MU** to the **public pool** and
- kept **80 MU** for his/herself

Group member 4 invested:
- **0 MU** into **his/her private pool**
- **40 MU** to the **public pool** and
- kept **60 MU** for his/herself

Hence, together, group member 1, 2, 3 and 4 invested **50 + 60 + 20 + 40 = 170 MU** into the **public pool**.

How many MU would group member 1 earn in this round?

0 10 20 30 40 50

How many MU would group member 2 earn in this round?

0 30 50 70 90 100

How many MU would group member 3 earn in this round?

0 10 20 30 40 50

How many MU would group member 4 earn in this round?

0 10 40 60 80 100

submit

comprehension check 4

Supplementary Figure 3. Comprehension check (continued).

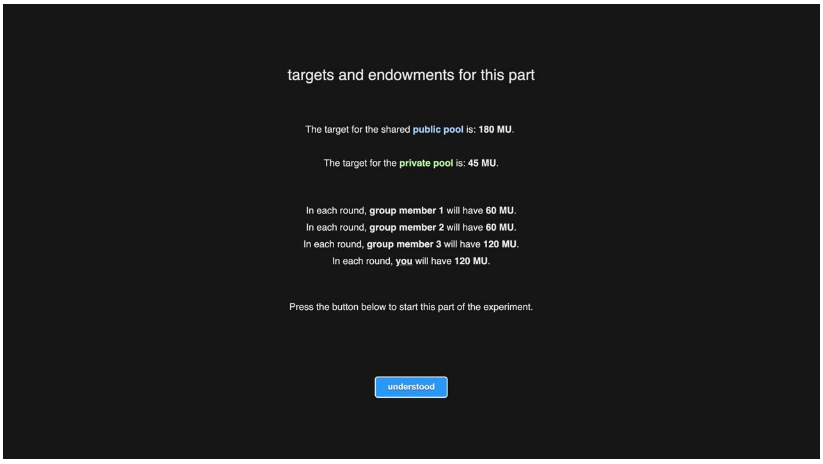

target announcement

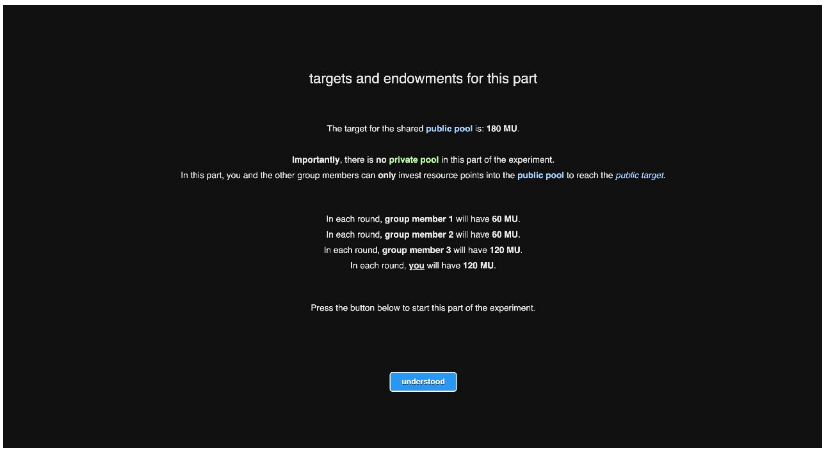

target announcement ($c_i = \infty$)

Supplementary Figure 4. Target announcement.

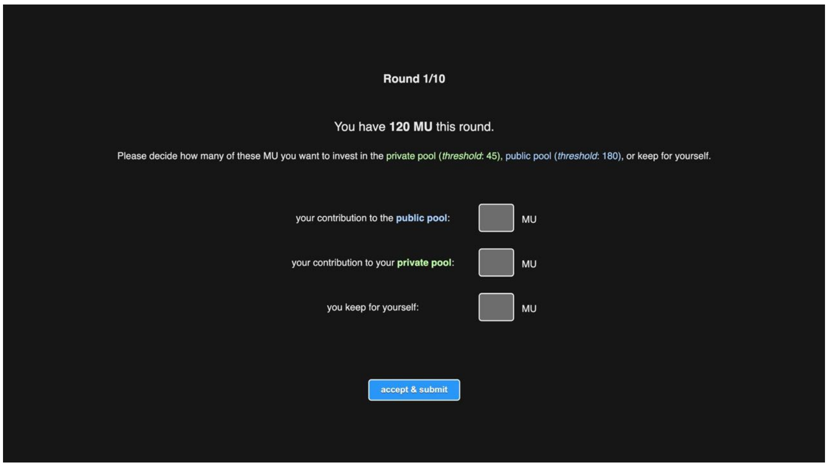

contribution stage

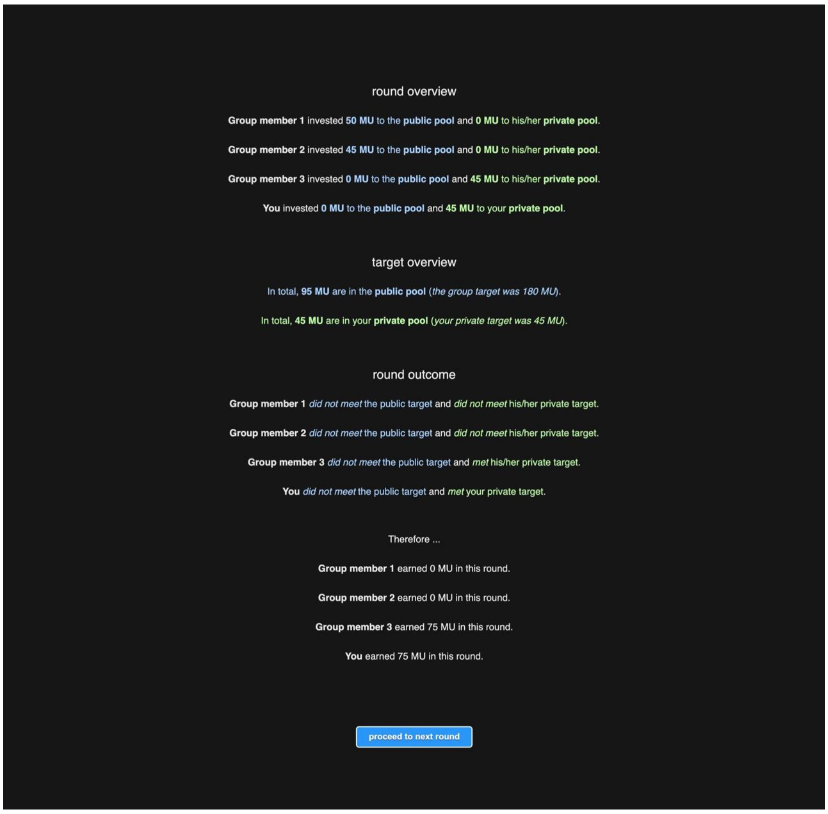

feedback stage

Supplementary Figure 5. Contribution and feedback stage.

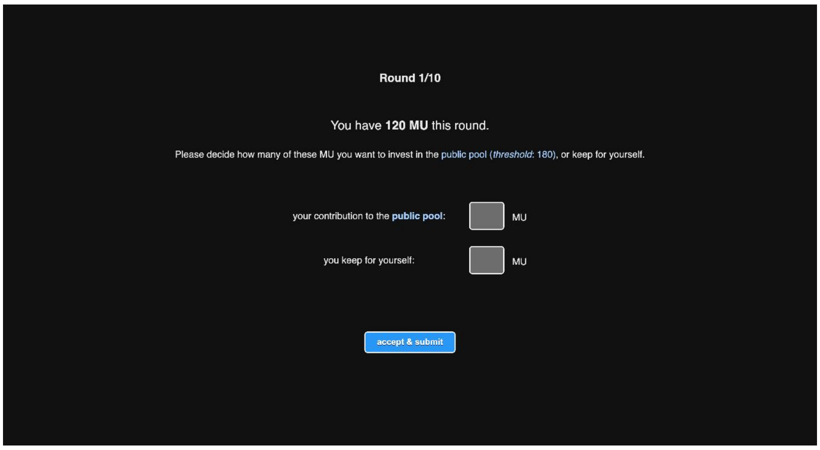

contribution stage ($c_i = \infty$)

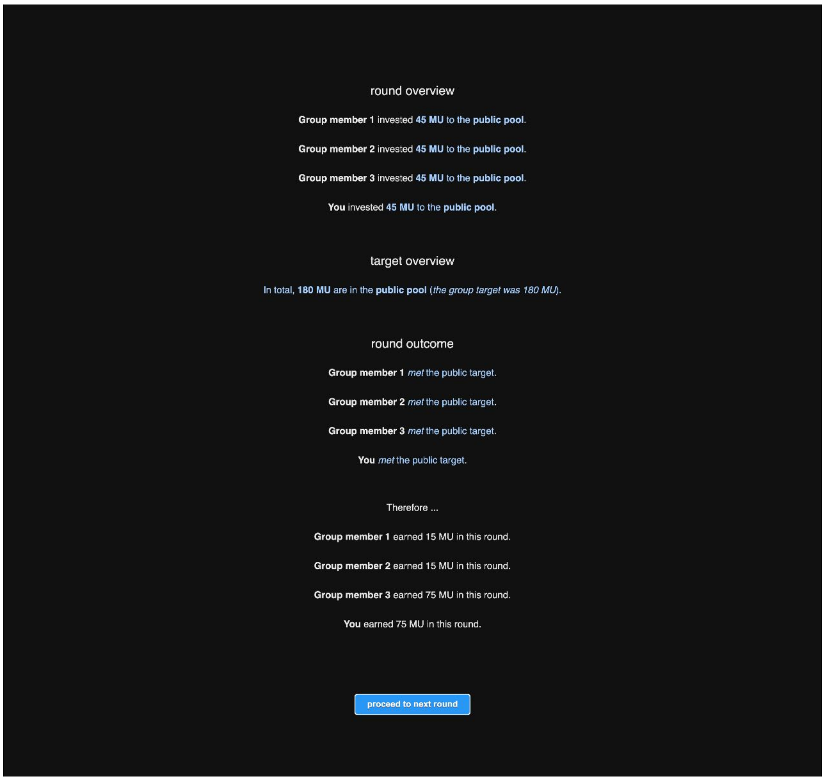

feedback stage ($c_i = \infty$)

Supplementary Figure 5. Contribution and feedback stage (continued).

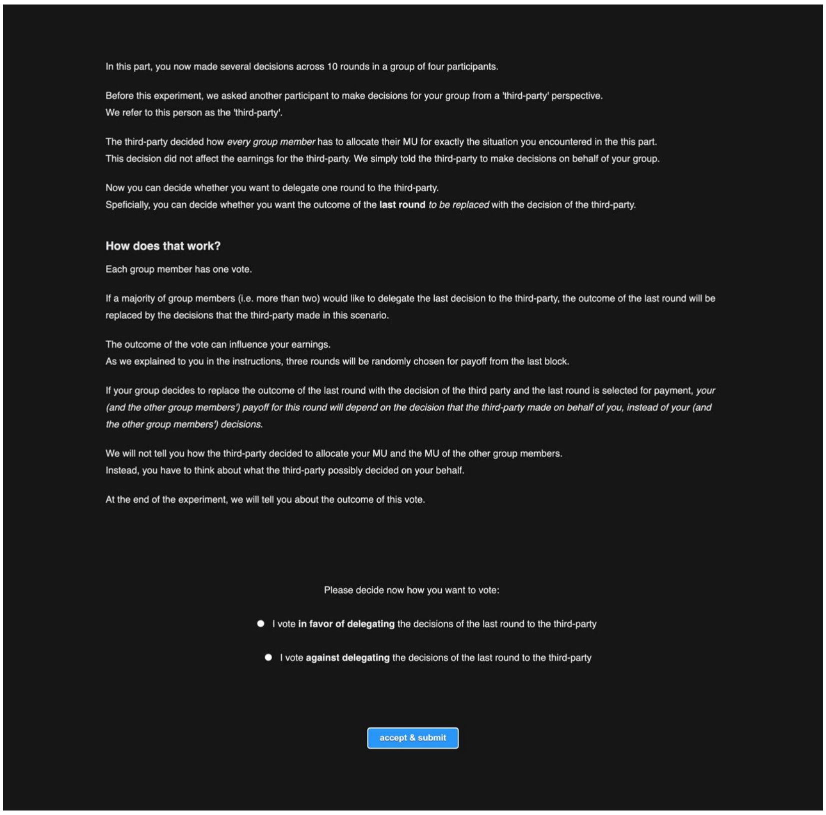

voting stage

Supplementary Figure 6. Voting stage.

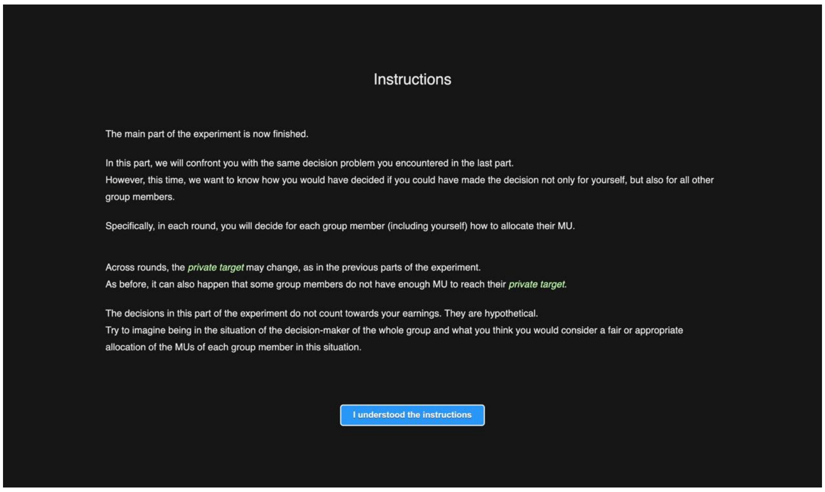

hypothetical 'dictator' decisions

Supplementary Figure 7. Fairness judgments.

Statistical Analysis

Because individual data-points (decisions) were clustered in individuals and groups, we either analysed data on the group-level to compare observations that satisfy the assumption of independence or fitted multilevel regression models as implemented in the lmer package in R (using the Satterthwaite's degrees of freedom method to derive p-values[15]). Results that were based on multilevel regressions are reported below in more detail. For some regressions, we were only interested in average group level effects across the dependence levels (c_i). In this case, we aggregated the data across groups and blocks and fitted a simple random intercept multilevel model (equation 1).

$$\begin{aligned} y_{jk} &= \beta_{0k} + \beta_1 X_{1jk} + e_{jk}, \;\; e_{jk} \sim N\left(0, \sigma_e^2\right) && \text{(level-1)} \\ \beta_{0k} &= \beta_0 + e_{0k}, \;\; e_{0k} \sim N\left(0, \sigma_{e_{0k}}^2\right) && \text{(level-2)} \qquad (1) \\ &\text{where } k = group, \;\; j = average\ response \end{aligned}$$

For more complex within-group comparisons (for example, dynamics over rounds or comparison between subjects within groups), we estimated two hierarchically clustered random intercepts to model responses (level 1) as nested in subjects (level 2) within groups (level 3), as shown in equation 2.

$$\begin{aligned} y_{ijk} &= \beta_{0jk} + \beta_1 X_{1ijk} + e_{ijk}, \;\; e_{ijk} \sim N\left(0, \sigma_e^2\right) && \text{(level-1)} \\ \beta_{0jk} &= \beta_{0k} + e_{0jk}, \;\; e_{0jk} \sim N\left(0, \sigma_{e_{0jk}}^2\right) && \text{(level-2)} \qquad (2) \\ \beta_{0k} &= \beta_0 + e_{0k}, \;\; e_{0k} \sim N\left(0, \sigma_{e_{0k}}^2\right) && \text{(level-3)} \\ &\text{where } k = group, \;\; j = subject, \;\; i = response \end{aligned}$$

For regressions that used c_i as a predictor, we coded interdependence as a continuous variable ranging from 0 (highest level of interdependence; $c_i = \infty$) to 4 (lowest level of interdependence; $c_i = 45$) when the data trend could be well approximated by a linear function of c_i. If that was not the case, we coded c_i as a factorial predictor (i.e. creating multiple dummy variables).

Since each group performed five different c_i blocks in a counterbalanced order (using the same counterbalance scheme across the symmetry and asymmetry condition), we also fitted each of the regression models reported below including either dummy variables that controlled for the specific c_i order of a group or dummy variables coding for the block-number (i.e. in which part

of the experiment a specific c_i level was encountered, which can also be interpreted as controlling for the experience with the game). None of the reported conclusions changed when adding these control variables. We, hence, report the simpler models below. All reported p-values below are two-sided (uncorrected if not otherwise noted).

Supplementary Note 1 – Models reported in the Main Manuscript

Creation of public vs. private goods across dependence levels (Figure 2)

Supplementary Table 1 shows the model results on how often groups successfully created public goods across the c_i levels on average (see Figure 2a in the main manuscript). For ease of interpretation and because the data trend could be well approximated by a linear decline (see Figure 2), we coded interdependence (operationalized as c_i cost) as a continuous variable ranging from 0 (Intercept baseline; $c_i = \infty$; highest interdependence) to 4 ($c_i = 45$; lowest interdependence). Coding interdependence levels as a categorical predictor yielded similar conclusions. Based on the model, introducing and continuously decreasing the cost of the private solution significantly reduced public goods creation by 12% per 10 RP cost reduction of the private solution in the asymmetry condition. Compared to the asymmetry condition, public goods creation deteriorated faster across cost levels in the symmetry condition (symmetry × cost level estimate). In the symmetry condition, decreasing the cost of the private solution by 10 RP reduced the frequency of solving the problem collectively by 21%.

Supplementary Table 2 shows the model results on how often groups created private goods across the c_i interdependence levels on average (see Figure 2b in the main manuscript). For private goods, we find the reverse pattern: Across c_i levels, contributions to private solutions increased by 14% in the asymmetry condition and by 23% in the symmetry condition.

Supplementary Table 1. Public goods creation.

Public goods creation (percentage of rounds in which the public threshold was met) as a function of the private solution cost ('dependence level', coded as 0: $c_i = \infty$, 1: $c_i = 75$, 2: $c_i = 65$, 3: $c_i = 55$, 4: $c_i = 45$) and compared to the symmetry condition.

**Coefficient**	**estimate**	**p**
Intercept ($c_i = \infty$; asymmetry condition)	0.79	<0.001
dependence level	-0.12	<0.001
symmetry condition	0.11	0.10
symmetry condition × dependence level	-0.09	<0.001
		**std. dev.**
$\sigma_{\text{level 1}}$		0.27
$\sigma_{\text{level 2}}$		0.17

Supplementary Table 2. Private goods creation.

Private goods creation (percentage of rounds in which the private threshold was met) as a function of the private solution cost ('dependence level', coded as 0: $c_i = \infty$, 1: $c_i = 75$, 2: $c_i = 65$, 3: $c_i = 55$, 4: $c_i = 45$) and compared to the symmetry condition.

**Coefficient**	**estimate**	**p**
Intercept ($c_i = \infty$; asymmetry condition)	-0.06	0.13
dependence level	0.14	<0.001
symmetry condition	-0.03	0.61
symmetry condition × dependence level	0.09	<0.001
		**std. dev.**
$\sigma_{\text{level 1}}$		0.23
$\sigma_{\text{level 2}}$		0.11

Wealth gap between less and more dependent group members (Figure 3c)

To investigate how private solution costs influence earnings and earnings disparity between less (e = 120 RP) and more dependent (e = 60 RP) group members, we calculated average relative earnings across rounds and types (less vs. more dependent group members) for each block and used the private solution cost level and group member type as predictors. Supplementary Table 3 shows that more dependent group members earned 4.1% less with increasingly cheaper private solutions. In contrast, less dependent group members earned 2.6% more with increasingly cheaper private solutions. In other words, the introduction of (increasingly cheaper) private solutions benefitted less dependent group members at the expense of more dependent group members.

Supplementary Table 3. Wealth-gap.

Earnings (as a percentage of starting endowment) as a function of the cost of private solutions ('dependence level', coded as 0: $c_i = \infty$, 1: $c_i = 75$, 2: $c_i = 65$, 3: $c_i = 55$, 4: $c_i = 45$) and RP in the asymmetry condition.

**Coefficient**	**estimate**	**P**
Intercept ($c_i = \infty$; e = 60 RP)	37.69	<0.001
dependence level	-4.08	<0.001
e = 120 RP	-4.60	0.07
dependence level × e = 120 RP	6.70	<0.001
		**std. dev.**
$\sigma_{\text{level 1}}$		11.38
$\sigma_{\text{level 2}}$		8.90
$\sigma_{\text{level 3}}$		6.80

Wealth inequality between conditions (Figure 3c Inset)

To compare the trajectory of inequality in earnings across c_i levels between the symmetry and asymmetry condition, we calculated the average Gini coefficient for each group and c_i block and entered it into multilevel regression with dependence level and condition as fixed-effects predictors. As can be seen in Supplementary Table 4, earnings inequality increased by 0.09

points in the asymmetry condition across the c_i parameter space. In contrast, changes in earnings inequality were significantly lower in the symmetry condition (symmetry condition × dependence level estimate) and only slightly increased by 0.03 points when private solutions became cheaper.

Supplementary Table 4. Wealth-inequality across conditions.

Inequality in earnings (Gini coefficient) as a function of the private solution cost ('dependence level', coded as 0: $c_i = \infty$, 1: $c_i = 75$, 2: $c_i = 65$, 3: $c_i = 55$, 4: $c_i = 45$) and condition.

**Coefficient**	**estimate**	**p**
Intercept ($c_i = \infty$; asymmetry condition)	0.21	<0.001
dependence level	0.09	<0.001
symmetry condition	-0.13	0.003
symmetry condition × dependence level	-0.06	<0.001
		**std. dev.**
$\sigma_{\text{level 1}}$		0.16
$\sigma_{\text{level 2}}$		0.08

Social preferences, cooperation, and welfare of more dependent group members (Figure 3d)

Supplementary Figure 8 shows the first-order associations between social preferences of less dependent group members and their cooperation rates (Supplementary Figure 8a, contributions to the public solution), and earnings of more dependent fellow group members depending on the average social preferences of their less dependent fellow group members (Supplementary Figure 8b) for $c_i \neq \infty$.

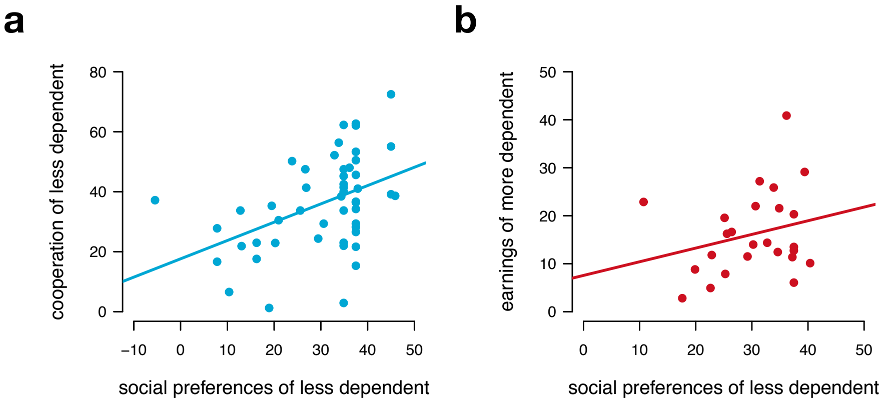

Supplementary Figure 8. Social preferences and cooperation. Association between social preferences of less dependent group members and their average cooperation (**a**) and earnings of more dependent group members (**b**) aggregated across $c_i \neq \infty$. Lines indicate best linear fit based on least-squares.

To understand how social preferences of less dependent group members relate to cooperative behaviour in the private-public goods dilemma and whether this relationship predicts the welfare of more dependent group members, we fitted a mediation model to our data. Specifically, we aggregated the data across blocks and types (less vs. more dependent group members) and computed the causal mediation model shown in Figure 3d. The relationship between SVO of less dependent group members and earnings of more dependent group members was fully mediated by the cooperativeness of less dependent group members. The standardized regression coefficient between pro-social preferences and contributions to the public solution of less dependent group members was statistically significant ($\beta = .43, P = 0.04$), as was the standardized regression coefficient between contributions to the public solution of less dependent group members and welfare of more dependent group members ($\beta = .79$, $P < 0.001$). The standardized indirect effect was (.43)(.79) = .34 ($P = 0.03$). There was no significant direct effect of pro-social preferences of less dependent group members and earnings of more dependent group members (standardized effect, $\beta_c = .22$, $P = 0.30$, standardized effect controlling for cooperation levels, $\beta_{c'} = -.12$, $P = 0.35$). In other words, the welfare of more dependent group members was conditional on the cooperation levels of less dependent group members, which were in turn predicted by their pro-social preferences. The model was fitted using the lavaan and mediation package in R. Significance was determined using bootstrapping (10,000 samples).

For the above analysis, we aggregated data across c_i levels. Yet, the relationship between pro-social preferences and cooperation also depended on the cost of private solutions c_i. Specifically, pro-social preferences of less dependent group members did not significantly

predict their cooperation levels under $c_i = \infty$ (b = -0.62, $t(66)$ = -0.65, P = 0.52), yet became a stronger predictor of cooperation as the cost of private solutions decreased (social preferences × private solution cost level, b = 1.13, $t(98)$ = 3.49, $P < 0.001$). In other words, only when private solutions were available, pro-social preferences predicted cooperation rates of less dependent group members. Consequently, the welfare of more dependent group members hinged on the pro-social concerns of less dependent group members in particular when (cheap) private solutions were available.

Supplementary Note 2 – Relative cooperation and failure likelihood across the c_i space

We ran additional analyses to supplement the reported results in the main manuscript and to gain additional insights into the behavioural dynamics of the private-public goods dilemma.

Supplementary Table 5 shows the effects of manipulating private solution costs on public goods provisions across and within groups. Under $c_i = \infty$, relative contribution rates did not differ between equally dependent group members vs. less or more dependent group members, respectively (e = 60 RP and e = 120 RP coefficients). Hence, when groups could only solve the problem collectively, dependence asymmetry did not alter relative cooperation levels. In the symmetry condition, relative contributions to the public pool steadily declined when private solutions became increasingly cheap (c_i coefficients). In comparison, relative cooperation rates of more dependent group members were significantly higher when introducing private solutions ($c_i \times e$ = 60 RP coefficients), while less dependent group members only increased their contribution levels significantly under $c_i = 45$ (compared to equally dependent groups; $c_i \times e$ = 120 RP coefficients). In other words, relative cooperation rates did not significantly differ between less, more, or equally dependent group members when there was no private solution. Compared to the symmetry condition, the introduction of private solutions 'forced' more dependent group members (e = 60) but not less dependent group members (e = 120) into contributing more of their resources to the public solution.

Supplementary Table 5. Cooperation across c_i and resource availability.

Cooperation (percentage of RP invested towards a public solution) as a function of the private solution cost and type (more dependent: e = 60 RP, equally dependent: e = 90 RP, less dependent: e = 120 RP).

**coefficient**	**estimate**	**p**
Intercept ($c_i = \infty$; e = 90 RP)	49.10	<0.001
c_i = 75 (e = 90 RP)	-1.12	0.56
c_i = 65 (e = 90 RP)	-14.67	<0.001
c_i = 55 (e = 90 RP)	-24.25	<0.001
c_i = 45 (e = 90 RP)	-45.27	<0.001
e = 60	-3.07	0.31
e = 120	3.00	0.33
c_i = 75 × e = 60 RP	7.35	0.03
c_i = 65 × e = 60 RP	25.11	<0.001
c_i = 55 × e = 60 RP	14.51	<0.001
c_i = 45 × e = 60 RP	25.67	<0.001
c_i = 75 × e = 120 RP	-6.45	0.05
c_i = 65 × e = 120 RP	5.49	0.10
c_i = 55 × e = 120 RP	4.73	0.15
c_i = 45 × e = 120 RP	17.60	<0.001
		**std. dev.**
$\sigma_{\text{level 1}}$		13.50
$\sigma_{\text{level 2}}$		6.46
$\sigma_{\text{level 3}}$		5.66

Likelihood of failure across the c_i space

Supplementary Table 6 shows the effects of manipulating private solution costs on the frequency of failure (i.e. earning 0 due to not reaching the public or private threshold) across and within groups. Under $c_i = \infty$, the likelihood of failure did not significantly differ between less or more dependent group members compared to equally dependent group members ($e = 60$ RP & $e = 120$ RP coefficients). Introducing private solutions to shared problems actually decreased the likelihood of failure in the symmetry condition (c_i coefficients). In the asymmetry condition, less dependent group members did not significantly differ from the failure rates of equally dependent group members when private solutions were available ($c_i \times e = 120$ RP coefficients). In stark contrast, failure frequency for more dependent group members increased significantly when introducing private solutions ($c_i \times e = 60$ RP coefficients, with the exception of $c_i = 55$ which only marginally differs from $c_i = 55$ for equally dependent groups). In other words, when private solutions were not available and the group only had an attainable public solution, having more or less resources did not significantly change the likelihood to lose everything. With the introduction of private solutions, more dependent group members ($e = 60$ RP) became increasingly likely to lose all resources because the problem was not (publicly) solved.

Supplementary Table 6. Failure likelihood.

Frequency of losing all remaining resources due to not meeting the private or public solution as a function of the private solution cost and type (more dependent: e = 60, equally dependent: e = 90, less dependent: e = 120).

coefficient	estimate	p
Intercept ($c_i = \infty$; e = 90 RP)	0.200	<0.001
c_i = 75 (e = 90 RP)	-0.072	0.01
c_i = 65 (e = 90 RP)	-0.014	0.62
c_i = 55 (e = 90 RP)	-0.050	0.07
c_i = 45 (e = 90 RP)	-0.129	<0.001
e = 60	0.068	0.13
e = 120	0.058	0.20
c_i = 75 × e = 60 RP	0.150	0.002
c_i = 65 × e = 60 RP	0.144	0.003
c_i = 55 × e = 60 RP	0.084	0.08
c_i = 45 × e = 60 RP	0.139	0.004
c_i = 75 × e = 120 RP	0.022	0.65
c_i = 65 × e = 120 RP	0.006	0.90
c_i = 55 × e = 120 RP	-0.030	0.53
c_i = 45 × e = 120 RP	0.015	0.76
		**std. dev.**
$\sigma_{\text{level 1}}$		0.20
$\sigma_{\text{level 2}}$		0.06
$\sigma_{\text{level 3}}$		0.10

Supplementary Note 3 – Opting-out and wealth consequences

Opting-out decisions

Supplementary Table 7 shows the estimated likelihood of not contributing to the public solution (i.e. $s_p = 0$) across (c_i levels) and within (RP endowment) groups (using RP = 60 as baseline). When private solutions were unavailable ($c_i = \infty$) group members did not differ in their likelihood of not contributing to the public pool ($e = 90$ & $e = 120$ coefficient compared to $e = 60$). Yet, with the introduction of private solutions, equally and less dependent group members had a significantly higher likelihood of choosing to not contribute anything to the public solution under all levels of c_i compared to more dependent group members ($c_i \times e = 90$ RP & $c_i \times e = 120$ RP coefficients) except for $c_i = 45$, for which less dependent group members only had a marginally significant higher likelihood to choose $s_p = 0$ compared to their fellow more dependent group members.

Supplementary Table 7. Opting out likelihood.

Binomial multilevel regression modelling the decision to not contribute anything to the public solution as a function of the private solution cost and type (more dependent: e = 60, equally dependent: e = 90, less dependent: e = 120).

Coefficient	estimate	p
Intercept ($c_i = \infty$; e = 60 RP)	-6.937	<0.001
c_i = 75 (e = 60 RP)	-0.536	0.47
c_i = 65 (e = 60 RP)	0.000	1.00
c_i = 55 (e = 60 RP)	4.851	<0.001
c_i = 45 (e = 60 RP)	6.379	<0.001
e = 90	-0.293	0.73
e = 120	-1.902	0.10
c_i = 75 × e = 90 RP	2.312	0.005
c_i = 65 × e = 90 RP	5.430	<0.001
c_i = 55 × e = 90 RP	1.757	0.006
c_i = 45 × e = 90 RP	3.678	<0.001
c_i = 75 × e = 120 RP	5.724	<0.001
c_i = 65 × e = 120 RP	5.477	<0.001
c_i = 55 × e = 120 RP	2.419	0.04
c_i = 45 × e = 120 RP	2.112	0.07
Round	0.104	<0.001

	std. dev.
$\sigma_{\text{level 2}}$	1.30
$\sigma_{\text{level 3}}$	1.63

Wealth gap between all three types

Supplementary Table 8 supplements the results reported in Supplementary Table 3 by adding the comparison to the symmetry condition. Under $c_i = \infty$, when private solutions were absent, more dependent group members (e = 60 RP) did not significantly differ in terms of relative earnings compared to equally dependent group members (e = 90 RP coefficient) and actually earned significantly more compared to less dependent group members (e = 120 RP coefficient). Yet, with the introduction and continuous reduction in the cost of private solutions, relative earnings increased for equally and less dependent group members (dependence level × e = 90 RP and dependence level × e = 120 RP coefficient), while they decreased for more dependent group members (private solution cost level coefficient).

Supplementary Table 8. Wealth-gap.

Earnings (as a percentage of starting RP) as a function of the dependence level (coded as 0: $c_i = \infty$, 1: $c_i = 75$, 2: $c_i = 65$, 3: $c_i = 55$, 4: $c_i = 45$) and RP.

coefficient	estimate	P
Intercept ($c_i = \infty$; e = 60 RP)	37.69	<0.001
dependence level	-4.08	<0.001
e = 90 RP	-0.32	0.90
e = 120 RP	-4.60	0.03
dependence level × e = 90 RP	4.96	<0.001
dependence level × e = 120 RP	6.70	<0.001

	std. dev.
$\sigma_{\text{level 1}}$	11.59
$\sigma_{\text{level 2}}$	5.61
$\sigma_{\text{level 3}}$	5.82

Supplementary Note 4 – Voting, third party decisions, and fairness judgements

Voting decisions

Supplementary Figure 9 shows the average voting pattern for less dependent and more dependent group members depending on the cost of the private solution together with the consequences of delegation in terms of earnings changes. Voting in favour of delegation significantly increased for more dependent group members across the c_i parameter space (multilevel binomial regression, $b = 0.20$, $P = 0.038$). Specifically, the odds to vote in favour of delegation increased by 22% for more dependent group members with every level of cost reduction of the private solution. Less dependent group members were generally less in favour of delegation (multilevel binomial regression, $b = -1.24$, $P = 0.013$). Independent of the c_i level, the odds to vote *against* delegation were 1/exp(-1.24) = 3.5 times higher for less dependent group members compared to more dependent group members. Across all c_i levels, the average support for delegation never exceeded 50% for less dependent group members (see Supplementary Figure 9, lower panel). There was no evidence for an interaction between participant type (less vs. more dependent) and c_i level (dependence level × type, $b = 0.13$, $P = 0.367$).

In the symmetry condition, group members also had the possibility to delegate one round to the third party after each block. On average, 32.4% of the equally dependent participants were in favour of delegation, which was significantly less compared to more dependent group members in the asymmetry condition ($e = 60$ vs. $e = 90$, two-sample t-test, $t(46) = -3.34$, $P = 0.001$) but did not significantly differ from the delegation support of less dependent group members in the asymmetry condition ($e = 120$ vs. $e = 90$, two-sample t-test, $t(38) = -0.67$, $P = 0.505$). There was no evidence that the support for delegation changed across c_i in symmetry groups (multilevel binomial regression, $b = -0.09$, $P = 0.197$). On average, symmetry groups had a majority in favour of delegation in 12% of the cases (compared to 30% in the asymmetry condition).

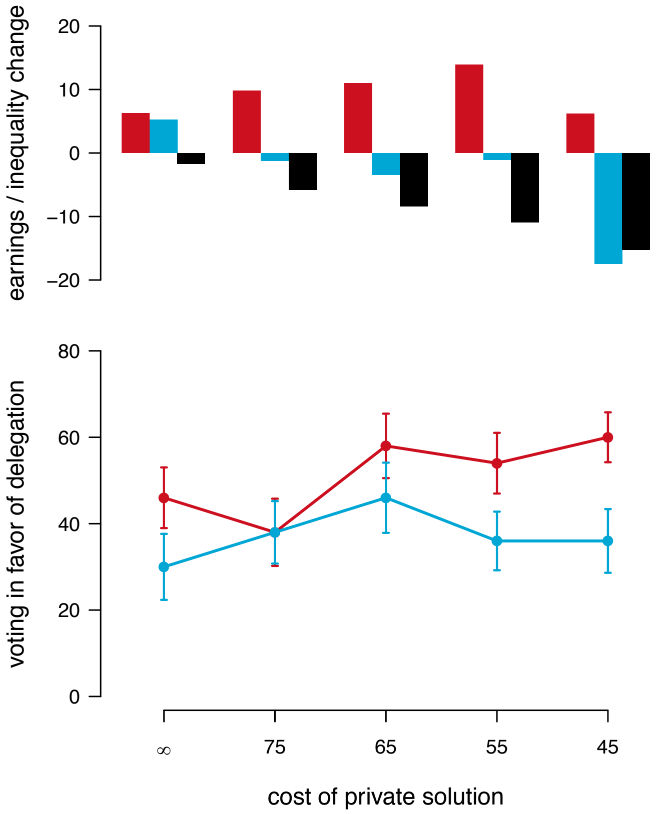

Supplementary Figure 9. Third-party delegation. Average voting decisions in favour of delegating the private-public goods problem to the third party for less dependent (n = 50, blue line) and more dependent (n = 50, red line) group members across the private solution cost c_i parameter space (lower panel). Upper panel: Consequences of delegation in terms of changes in earnings for less dependent (blue/middle bars) and more dependent (red/left bars) group members and changes in earnings disparity (measured as the standard deviation in earnings, black/right bars) compared to the actual outcome. Points and bars indicate the mean. Error bars indicate the standard error of the mean.

Third Party decisions

Third-party decision makers solved the group's problem predominantly by creating collective solutions and significantly more than the actual groups (Supplementary Figure 10a, multilevel regression, symmetry condition: third party vs. groups [$c_i = \infty$], $b = 0.10$, $P = 0.153$, third party × dependence level, $b = 0.12$, $P < 0.001$; asymmetry condition: third party vs. groups [$c_i = \infty$], $b = 0.18$, $P = 0.005$, third party × dependence level, $b = 0.09$, $P < 0.001$). Conversely, third parties created fewer private solutions (Supplementary Figure 10b, multilevel regression, symmetry condition: third party × dependence level, $b = -0.13$, $P < 0.001$; asymmetry condition: third party × dependence level, $b = -0.09$, $P = 0.001$).

Average group earnings were also significantly higher when third parties made the decisions for the group in both the symmetry (Supplementary Figure 10c, multilevel regression, third party vs. groups main effect, $b = 8.38$, $P = 0.004$) and asymmetry condition (multilevel regression, third party vs. groups main effect, $b = 10.16$, $P = 0.001$), except for $c_i = 45$. Lastly, third parties invested resources such that earnings were more equally distributed among group members (Supplementary Figure 10d). In the asymmetry condition, the earnings disparity was significantly lower under all private solution costs when third parties made the decision (multilevel regression, third party × $c_i = 75$, $b = -0.11$, $P = 0.049$, third party × $c_i = 65$, $b = -0.20$, $P < 0.001$, third party × $c_i = 55$, $b = -0.23$, $P < 0.001$, third party × $c_i = 45$, $b = -0.27$, $P < 0.001$; third party vs. groups [$c_i = \infty$], $b = -0.01$, $P < 0.778$). In the symmetry condition, earnings disparity was only significantly lower when third parties made decisions for $c_i = \{65,55\}$ (multilevel regression, third party × $c_i = 75$, $b = -0.09$, $P = 0.068$, third party × $c_i = 65$, $b = -0.21$, $P < 0.001$, third party × $c_i = 55$, $b = -0.13$, $P = 0.006$, third party × $c_i = 45$, $b = -0.02$, $P = 0.683$; vs. third party vs. groups [$c_i = \infty$], $b = 0.00$, $P = 0.914$).

Fairness judgments

After the main experiment, we asked group members how they would allocate the resources of all group members, if they would have the power to do so. This allowed us to probe redistribution preferences. There are two relevant fairness heuristics for creating a public solution under unequal resource distribution[16,17]. According to an 'equality in contributions rule', each group member should contribute 50% of their resources. According to an 'equality in outcomes rule', less dependent group members should contribute 75% and more dependent group members should contribute 37.5% of their resources, resulting in each group member being left with 45 RP (Supplementary Figure 11a). When asked, both less and more dependent group members indicated to favour a resource allocation more akin to the equality in outcomes rule for the creation of public goods, similar to progressive taxation (aggregated across c_i levels, Supplementary Figure 11b). In the experiments, however, group behaviour was more akin to the equality in contributions rule, similar to flat taxation (Supplementary Figure 11c). Hence, both less and more dependent group members indicated to prefer a redistribution of resources such that less dependent group members would bear a higher cost of creating public solutions. This was both at odds with what happened in the actual dilemma situation (Supplementary Figure 11c) as well as with the voting pattern on delegating decisions to third parties, suggesting that fairness rules did not drive behaviour. Likely, less dependent group members express to be in favour of fair redistribution when these expressions are non-consequential and, thus, 'cheap'.

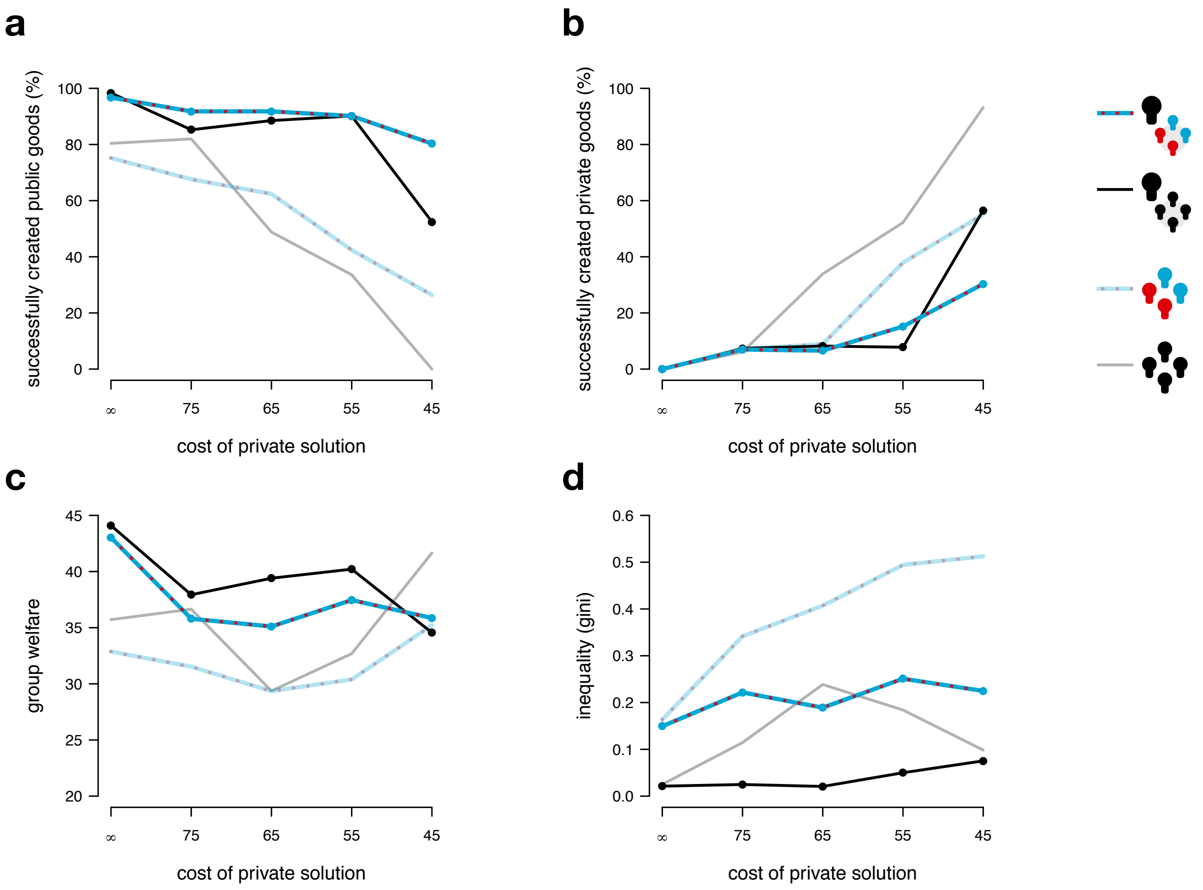

Supplementary Figure 10. Third party behaviour. Average public goods creation (**a**), private goods creation (**b**), achieved group welfare (i.e. average group earnings) (**c**), and welfare inequality (as measured by the Gini coefficient) (**d**) for third parties ($n = 61$) making decisions on behalf of asymmetry groups (blue-red line) and symmetry groups (black line) in comparison to the actual average outcome of asymmetry groups ($n = 25$, transparent blue-red line) and symmetry groups ($n = 25$, transparent black line). Points indicate the mean.

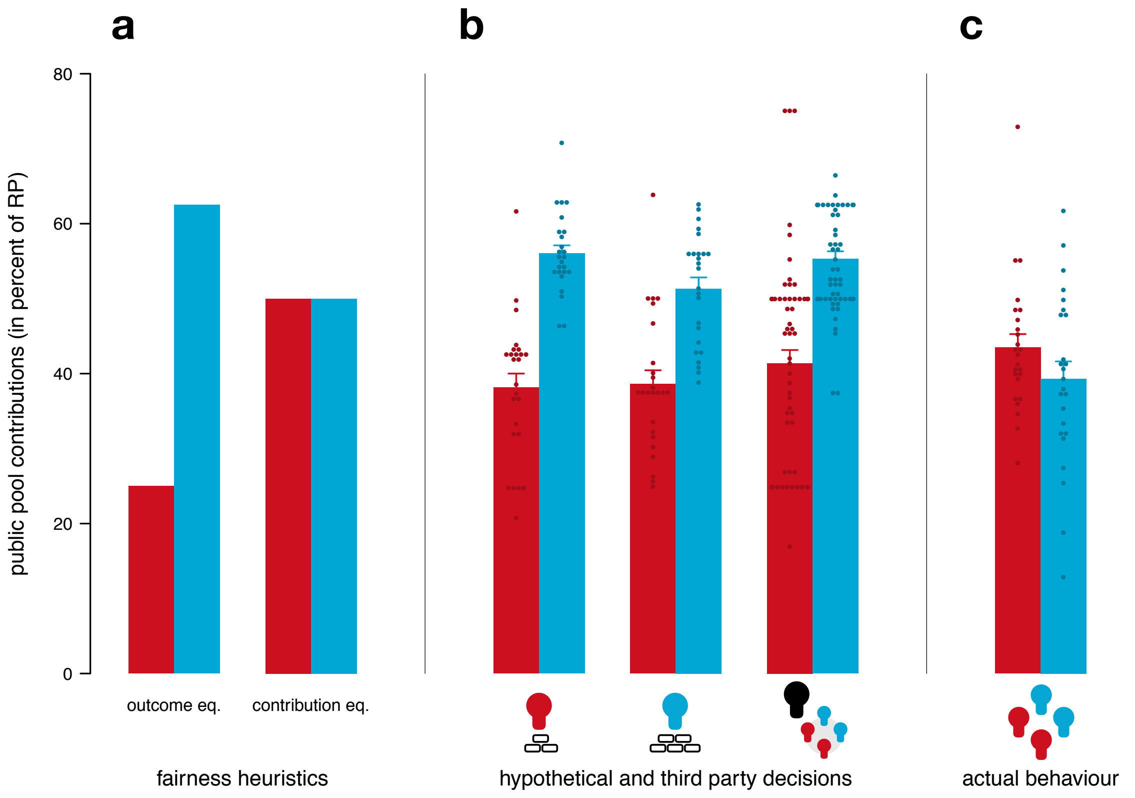

Supplementary Figure 11. Fairness perceptions. Groups in the asymmetry condition can solve the problem collectively by aiming for outcome equality, in which case ex ante inequality in resources between group members would be nullified, or for contribution equality, in which case everyone contributes 50% of their endowment (**a**). In hypothetical decisions, both more (n = 50, red) and less dependent (n = 50, blue) group members indicated that, on average, they favoured an allocation of resources that was more in line with the outcome equality benchmark. Their stated preferences were similar to how the third-party decision makers (n = 61) actually distributed resources on behalf of the group (**b**). The average public pool contributions in the experiment were, however, closer to the contribution equality benchmark (**c**). Points indicate individual data points. Bars indicate the mean. Error bars indicate the standard error of the mean.

Supplementary Note 5 – Taking advantage of asymmetric access to private solutions

In the asymmetry condition, we modelled a situation in which group members, across private solution cost levels, differ in their dependence on others' cooperation. At the same time, group members differed in their ability to be self-reliant. This allows us to contrast three different situations: Under $c_i = \infty$, all group members depend on cooperation. Under $c_i = \{75,65\}$, a private solution is only affordable for less dependent group members. Lastly, under $c_i = \{55,45\}$, all group members have access to a private solution. Hence, the ability to be self-reliant is 'impossible for all' with $c_i = \infty$, 'exclusive' (i.e. only affordable by less dependent group members) with $c_i = \{75,65\}$, and 'possible for all' with $c_i = \{55,45\}$.

Especially when private solutions are exclusive, less dependent group members can resort to private solutions while more dependent group members cannot. Theoretically, less dependent group members have more 'power' over more dependent group members in this situation, because more dependent group members have to rely on some support of less dependent group members which is not the case the other way around. This should enable less dependent group members to 'coerce' more dependent group members into higher levels of cooperation. When self-reliance is 'possible for all', this strict power asymmetry disappears, because less dependent group members can resort to become self-reliant and, strictly speaking, cannot be forced into higher levels of cooperation.

To test these predictions, we first looked at average contribution rates to the public solution across these three dependence-(a)symmetry levels ('impossible for all', 'exclusive', and 'possible for all'). When private solutions were unavailable, less dependent group members actually contributed a higher proportion of their RP to the public solution compared to more dependent group members (Supplementary Figure 12 & Supplementary Table 9, e = 60 RP coefficient). This reversed when private solutions were exclusively available for less dependent group members. Less dependent group members *reduced* their contribution to the public solution by 8.4%, while more dependent group members *increased* their contribution to the public solution by 8.3% (Supplementary Figure 12 & Supplementary Table 9, exclusive coefficient & exclusive × e = 60 RP coefficient). When private solutions were available for all, cooperation rates of less dependent and more dependent group members converged again. Less dependent group members dedicated 28.5% of their resources to the public solution, while more dependent group members did not assign significantly more of their resource to the public solution on average (31.4%, comparison: e = 60 RP vs. e = 120 RP when $c_i = \{55,45\}$, $b = 2.85$, $P = 0.35$).

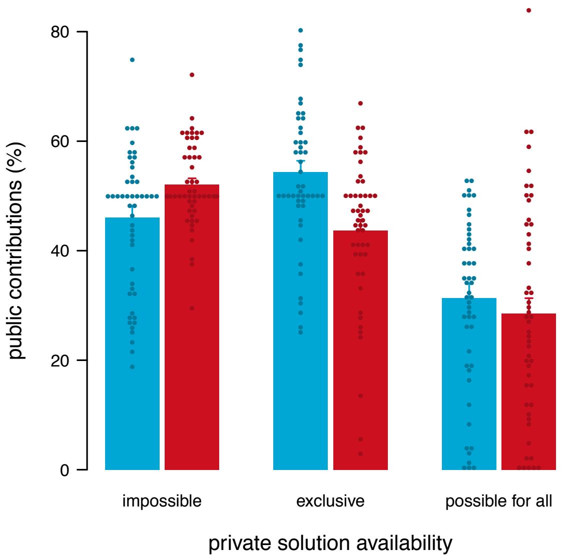

Supplementary Figure 12. Cooperation rates depending on private solution availability. Relative contributions to the public solution for less dependent (n = 50, blue/left bars) and more dependent group members (n = 50, red/right bars) when private solutions are impossible for all ($c_i = \infty$), exclusively attainable for less dependent group members ($c_i = \{75,65\}$), or possible for all ($c_i = \{55,45\}$). Points indicate individual data points. Bars indicate the mean. Error bars indicate the standard error of the mean.

Within groups, the change in the cooperation rates of more dependent group members was indeed correlated with the change in cooperation of less dependent group members in line with the above outlined predictions. To see that, we calculated the relative change in cooperation (%RP contributed to the public pool) when private solutions were not available ($c_i = \infty$) vs. when private solutions were exclusively available for less dependent group members or available for all group members. Supplementary Figure 13a shows that changes in cooperation levels by less dependent group members were negatively correlated with changes in cooperation levels by more dependent group members when private solution were exclusively available (Spearman r = -0.74, P < 0.001). In other words, the more group members with e = 120 decreased their cooperation when moving from $c_i = \infty$ to $c_i = \{75,65\}$, the more group members with e = 60 increased their cooperation. Yet, when private solutions were not exclusive anymore, this relationship reversed (Supplementary Figure 13b). The more group members with e = 120 decreased their cooperation when moving from $c_i = \infty$ to $c_i = \{55,45\}$, the more group members with e = 60 also decreased their public pool contributions (Spearman r = 0.57, P = 0.003). One interpretation of this finding, along the lines of the above argument, is that lower cooperation rates by less dependent group members 'forces' more dependent group members into higher levels of cooperation when private solutions are only available to them.

When private solutions are available for all group members, however, more dependent group members can ‘respond’ to lower levels of cooperation by less dependent group members by becoming self-reliant themselves. Yet, since private solutions do not allow any form of redistribution (every group member has to pay the cost herself), inequality perpetuates.

Supplementary Table 9. Cooperation across private solution availability.

Cooperation (percentage of RP invested towards a public solution) as a function of the private solution availability ($c_i = \infty$: impossible for all; $c_i = \{75,65\}$: exclusively available for less dependent group members; $c_i = \{55,45\}$: possible for all).

**coefficient**	**estimate**	**p**
Intercept ($c_i = \infty$; e = 120 RP)	52.10	<0.001
e = 60 RP ($c_i = \infty$)	-6.08	0.048
exclusive (e = 120 RP)	-8.38	<0.001
possible for all (e = 120 RP)	-23.59	<0.001
exclusive × e = 60 RP	16.72	<0.001
possible for all × e = 60 RP	8.93	0.0121
		**std. dev.**
$\sigma_{\text{level 1}}$		12.46
$\sigma_{\text{level 2}}$		8.86
$\sigma_{\text{level 3}}$		1.45

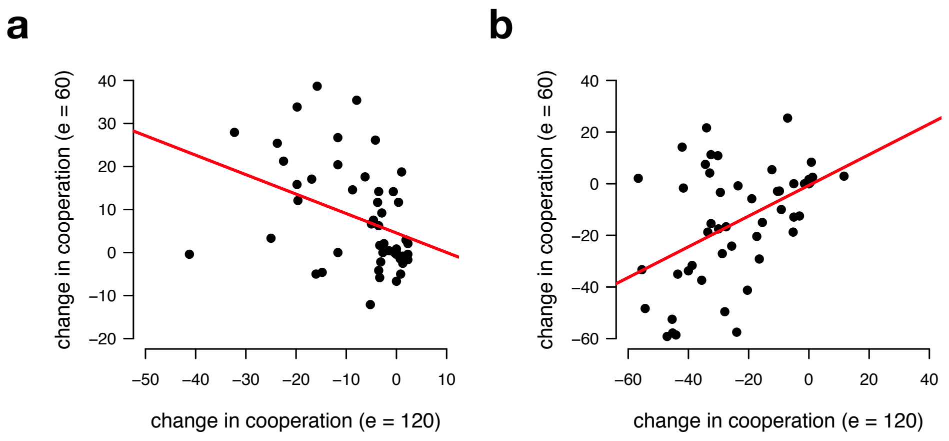

Supplementary Figure 13. Change in cooperation across less dependent and more dependent group members. When private solutions are exclusively attainable for less dependent group members ($c_i = \{75,65\}$) the change in cooperativeness of less dependent group members (n = 50), compared to $c_i = \infty$ (x-axis), is negatively correlated with the change in cooperativeness of more dependent group members (n = 50) (**a**). When private solutions are attainable to all (c_i = {55,45}), the reverse is true: the change in cooperativeness of less dependent group members, compared to $c_i = \infty$ (x-axis), is positively correlated with the change in cooperativeness of more dependent group members. Lines indicate best linear fit based on least-squares.

Private solutions as a form of punishment

For group members with e = 120 it is relatively cheaper to solve the shared problem individually – they do not depend on public solutions as much as their fellow group members with e = 60. From this perspective, solving the problem individually can also be seen as a way to punish other group members or signal that they should increase their public contributions. To analyse whether group members with e = 120 use the private solution strategically as a form of signalling or punishment device, we first checked whether the likelihood to meet the private threshold for group members with e = 120 was contingent on the public contributions of group members with e = 60 in the previous round. Supplementary Figure 14a shows that indeed, the lower the public contributions of group members with e = 60 in the previous round, the higher the likelihood that a group member with e = 120 would meet her private target. To test this more formally, we regressed the likelihood to meet the private target for group members with e = 120 on the average public contributions of group members with e = 60 in the previous round, controlling for own contributions in the previous round, contribution to the public pool in the present round, and the c_i level. Importantly, we only selected rounds in which the deciding group member did *not* meet her private target in the last round. Hence, the predictors indicate

the likelihood of a group member with $e = 120$ to switch from not meeting the private threshold in the previous round to meeting the private threshold in the present round. The regression model revealed that for every point decrease in contributions to the public pool by group members with $e = 60$, the likelihood to withdraw support for a public solution and instead meet the private target increased by 7% (multilevel regression, $b = -0.1105$, $P < 0.001$). This suggests that group members with $e = 120$ use the private solution as a punishment device or a means to signal to more dependent group members that they should contribute more to a public solution if they want their support.

To test whether such behaviour actually influenced the behaviour of more dependent group members, we first looked at the average public contributions of group members with $e = 60$ when group members with $e = 120$ did vs. did not meet their private target in the previous round (Supplementary Figure 14b). Indeed, public contributions of group members with $e = 60$ were positively related to the number of group members with $e = 120$ that met their private target in the previous round. To test this more formally, we regressed public pool contributions of more dependent group members ($e = 60$) on the number of group members with $e = 120$ that met their private target in the previous round. Based on the results of the previous section on cooperation (which is negatively related to private contributions), it should follow that the 'coercive' power of meeting the private threshold depends on whether more dependent group members actually have a (albeit) costly private alternative available or not. Indeed, when more dependent group members could not afford the private solution ($c_i = \{75,65\}$), we found evidence that they increased their public pool contributions the more group members with $e = 120$ met their private target in the previous round (multilevel regression, $b = 13.42$, $P < 0.001$). This relationship reversed (see also Supplementary Figure 13), when more dependent group members had a 'way out' ($c_i = \{45,55\}$; multilevel regression, $b = -7.935$, $P < 0.001$).

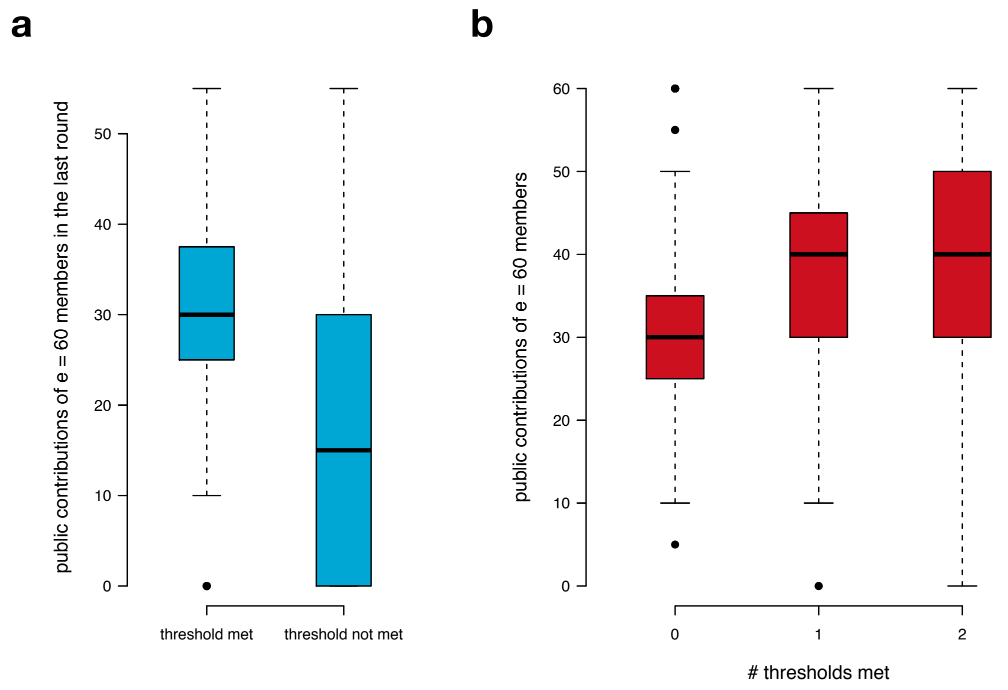

Supplementary Figure 14. Self-reliance as a form of punishment. (**a**) The prevalence of less dependent group members (*e* = 120, *n* = 50) meeting their private threshold was associated with lower average public contributions of more dependent group members (*e* = 60, *n* = 50) in the previous round (**b**). Less dependent group members' (*e* = 60) cooperation increased, the more group members with *e* = 120 (y-axis) met their private threshold in the previous round. Center line indicates the median; box limits indicate upper and lower quartiles; whiskers indicate the 1.5 interquartile range; points indicate outliers.

Supplementary Note 6 – Time dynamics

Dynamics over rounds

Supplementary Figure 15 shows the trajectory of public goods creation and inequality across rounds separately for each level of the private solution cost. When private solutions were not available ($c_i = \infty$), successful coordination on a public solution significantly increased over rounds, both in the symmetry condition (Supplementary Figure 15a, red line, multilevel binomial regression, $b = 0.42$, $P = 0.003$, all reported tests Bonferroni corrected for multiple comparison) and asymmetry condition (Supplementary Figure 15b, red line, multilevel binomial regression, $b = 0.35$, $P = 0.007$). Coordination on public solutions also significantly increased in the symmetry condition with $c_i = 75$ (Supplementary Figure 15a, yellow line, multilevel binomial regression, $b = 0.60$, $P < 0.001$) but not in the asymmetry condition (Supplementary Figure 15b, yellow line, multilevel binomial regression, $b = 0.05$, $P = 1$). Likewise, there was a significant increase in the successful creation of public goods in the symmetry condition with $c_i = 65$ (Supplementary Figure 15a, blue line, multilevel binomial regression, $b = 0.35$, $P = 0.009$) but not in the asymmetry condition (Supplementary Figure 15b, blue line, multilevel binomial regression, $b = 0.18$, $P = 0.187$). With $c_i = 55$ and $c_i = 45$, there was no significant change over time in either condition (Supplementary Figure 15ab, purple and green line, all $P > 0.23$). Hence, when private solutions were not available, groups in both conditions learned to more frequently coordinate on public solutions. When private solutions were rather expensive ($c_i = \{75,65\}$), symmetry groups ($e = 90$ RP) also increasingly coordinated on the public solution. On the flipside, the frequency of solving the shared problem cooperatively did not significantly change over time when private solutions were available in the asymmetry condition.

In the symmetry condition, inequality in earnings did not significantly change under $c_i = \infty$ (Supplementary Figure 15c, red line, multilevel regression, $b = 0.00$, $P = 1$), but significantly reduced over rounds with $c_i = 75$ (Supplementary Figure 15c, yellow line, multilevel regression, $b = -0.03$, $P = 0.002$), $c_i = 65$ (Supplementary Figure 15c, blue line, multilevel regression, $b = -0.04$, $P < 0.001$), $c_i = 55$ (Supplementary Figure 15c, purple line, multilevel regression, $b = -0.03$, $P = 0.001$), and $c_i = 45$ (Supplementary Figure 15c, green line, multilevel regression, $b = -0.03$, $P < 0.001$). In comparison, in the asymmetry condition, inequality over rounds changed significantly only with $c_i = 45$ (Supplementary Figure 15d, green line, multilevel regression, $b = -0.03$, $P < 0.001$). Across all other c_i levels, there was no significant time trend in the asymmetry condition (Supplementary Figure 15d, all $P > 0.26$). Hence, in symmetrically

dependent groups, inequality reduced over rounds when private solutions were available. In groups with dependence asymmetry, earnings inequality remained rather high and stable.

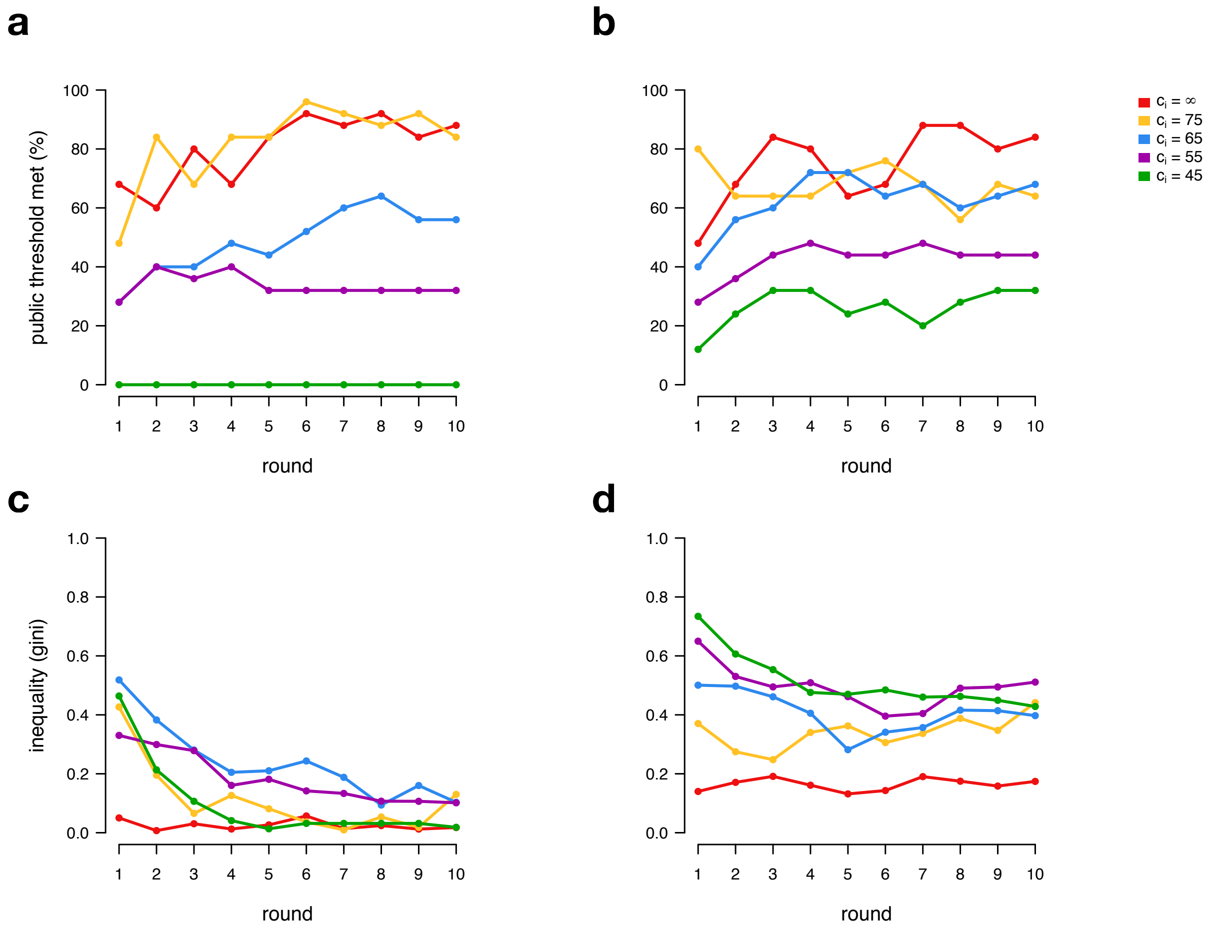

Supplementary Figure 15. Dynamics over rounds. Average percentage of public thresholds met (upper panels) in the symmetry (**a**) and asymmetry condition (**b**). Average within-group inequality in earnings (measured by the Gini coefficient, lower panels) in the symmetry (**c**) and asymmetry condition (**d**). Red line: $c_i = \infty$, yellow line: $c_i = 75$, blue line: $c_i = 65$, purple line: $c_i = 55$, green line: $c_i = 45$. Points indicate the mean.

Dynamics over blocks

Each group faced the private-public goods game across five blocks of 10 rounds with different c_i levels. On the one hand, a repeated measures design allows to investigate dynamics over time and analyse how the same groups change their behaviour across different incentive schemes. On the other hand, groups gain experience with the task that may also change their behaviour over time. To analyse changes over time, independent of the incentives introduced by changing the cost of self-reliance, we further analysed decision time patterns and variance in cooperation across rounds and blocks.

Supplementary Figure 16 shows the average response time across blocks and rounds. In general, response time decreased over rounds, converging to an average of around 7.6 seconds per decision. Average decision time was significantly higher in the first block compared to the other blocks (multilevel regression, all $P < 0.001$) and average decision time in the second block was significantly higher than in the other blocks (multilevel regression, all $P < 0.001$), while average decision time did not significantly deviate in block three to five. This shows that participants made faster decisions with more experience in the task. Note that decision time should be interpreted with some caution in the first rounds of the first block. While participants were encouraged to ask questions reading the instructions and comprehension check part, they may still have contacted the experimenter for questions at the beginning of the actual experiment, which we cannot control for but alters the interpretation of this measure.

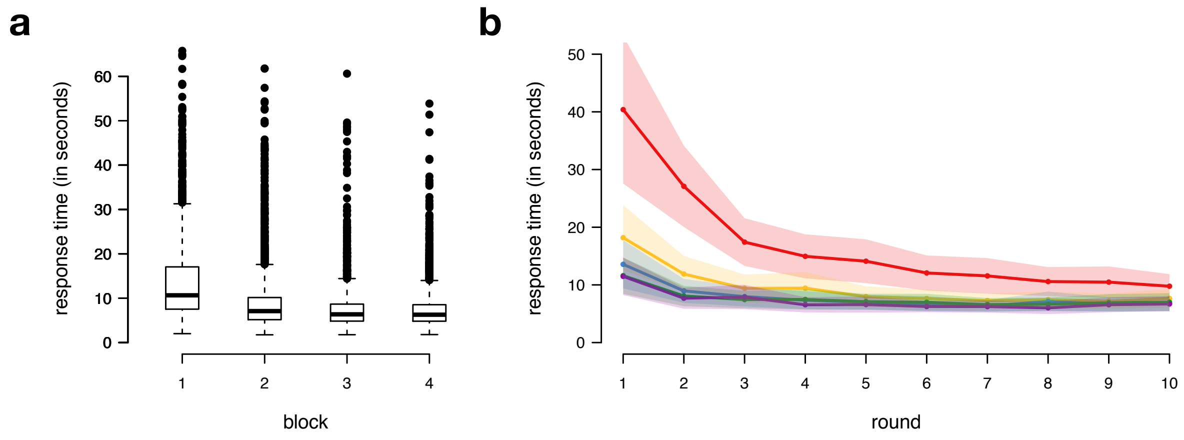

Supplementary Figure 16. Change in decision time across blocks. Distribution of decision time across blocks (**a**) and average decision time across rounds per block (**b**, red = first block, yellow = second block, blue = third block, green = fourth block, purple = fifth block). Based on $n = 200$; Center line indicates the median; box limits indicate upper and lower quartiles; whiskers indicate the 1.5 interquartile range; points indicate outliers. Points indicate the mean. Confidence bands indicate the standard error of the mean.

The decision time pattern suggests that, with more experience, participants take less time to decide. Supplementary Figure 17 further shows that participants also changed their cooperation decisions across rounds more frequently in the first five rounds compared to the last five rounds in each block and more so in the first block than the other blocks. This was particularly true in the symmetric condition (Supplementary Figure 17a), showing that participants converged to a stable state that, once reached, may have required less time to decide. In the asymmetric condition, in comparison, the within-subject variance in cooperation decision stayed high and dropped less sharply between the first five rounds and last five rounds of each block

(Supplementary Figure 17b), suggesting that in the asymmetric condition, behaviour was less stable and participants did not converge to a stable decision pattern as much. This pattern resonates with the dynamics over rounds in meeting the public threshold (see above) and the results on taking advantage of asymmetric availability of private solutions. In general, the situation seems more dynamic in the asymmetric condition, meaning that reactions and counter-reactions to the behaviour of other group members lead to more fluctuation in the own decision to cooperate.

Note that we counterbalanced block-order across groups and conditions. Additional regressions controlling for the block number (as a measure for experience with the game) showed that reported results remained robust to these controls.

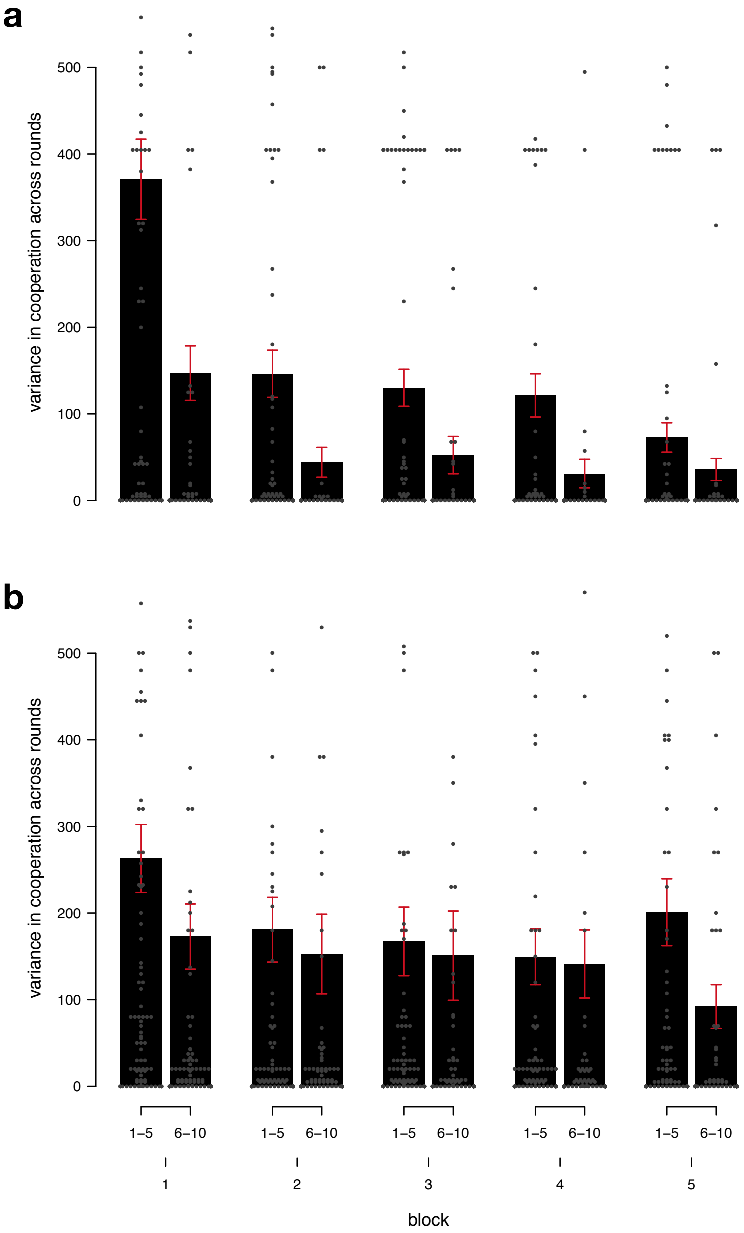

Supplementary Figure 17. Change in cooperation decisions across rounds and blocks. Average within-subject variance in cooperation, as a measure of choice consistency, in the first five rounds and last five rounds of every block in the symmetry condition (n = 100) (**a**) and asymmetry condition (n = 100) (**b**). Points indicate individual data points. Bars indicate the mean. Error bars indicate the standard error of the mean.

Supplementary Note 7 – Cooperation and interindividual difference measures

For each participant, we obtained risk preferences, social preferences, and demographic information (age and sex) that could explain interindividual differences in cooperation depending on whether private solutions were available or not. For example, in the symmetry condition, social preferences (as measured by the social value orientation slider measure) should be correlated with cooperation under $c_i = \infty$ (i.e. when there is no private alternative and the game reduces to a step-level public goods game), but may be less correlated with social preferences when private solutions are available (see [18] for a related finding). Further, risk-aversion should be correlated with cooperation under $c_i = \infty$, since high cooperation rates reduce the risk of not meeting the public threshold and lose all remaining resources. In the asymmetric condition, the relation of cooperation and social preferences may be more complex. For example, when private solutions are available, members with $e = 120$ may still opt to contribute to the public pool because of a concern for members that are less able to become self-reliant (as already shown above).

To analyse the association of interindividual difference measures with cooperation, we regressed social preferences, risk aversion (measured as the number of times a participant chose the safe rather than the risky option in our risk measure), age, and sex on average contributions to the public pool separate for each agent type ($e = 60$, $e = 90$, $e = 120$) and separately for when a private solution was attainable for the participant or not.

As can be seen in Supplementary Table 10 & 11 and Supplementary Figure 18, social value orientation (i.e. social preferences) was the only variable that was significantly associated with cooperation rates in the symmetry condition when private solutions were not attainable. However, the association between social preferences reduced when private solutions were available (Supplementary Figure 18) and did not significantly predict cooperation rates in the regression model anymore (Supplementary Table 11), suggesting that social value orientation can explain interindividual differences in cooperation in the classic cooperation dilemma, but less so in the social dilemma of self-reliance.

Supplementary Table 10. Interindividual differences and cooperation (e = 90).

Average cooperation (RP invested towards a public solution) as a function of interindividual difference measures when private solutions were not attainable ($c_i = \infty$).

coefficient	estimate	p
Intercept	36.96	<0.001
social value orientation	0.21	<0.001
risk aversion	3.11	0.406
age	0.00	1.000
sex (0 = female, 1 = male)	-2.28	0.116
		**std. dev.**
$\sigma_{\text{level 1}}$		6.00
$\sigma_{\text{level 2}}$		0.00

Supplementary Table 11. Interindividual differences and cooperation (e = 90).

Average cooperation (RP invested towards a public solution) as a function of interindividual difference measures when private solutions were attainable ($c_i \neq \infty$).

coefficient	estimate	p
Intercept	17.46	<0.001
social value orientation	0.09	0.063
risk aversion	1.15	0.735
age	0.17	0.290
sex (0 = female, 1 = male)	2.58	0.054
		**std. dev.**
$\sigma_{\text{level 1}}$		4.60
$\sigma_{\text{level 2}}$		7.30

Interestingly, for participants with *e* = 120 in the asymmetry condition, we observe the reverse: When the shared problem could only be solved collectively ($c_i = \infty$), social preferences were only weakly associated with cooperation rates (Supplementary Table 12, see also Supplementary Figure 15), while they were significantly correlated with cooperation rates when private solutions were available for the decision maker (Supplementary Table 13, see also Supplementary Figure 18). Arguably, in the asymmetry condition, choosing to opt for the private solution is perceived as an action with more pronounced social consequences for others compared to the symmetry condition, since not every group member has the same ability to be self-reliant. Hence, social preferences play a more important role in this situation (see also mediation results above).

Supplementary Table 12. Interindividual differences and cooperation (e = 120).

Average cooperation (RP invested towards a public solution) as a function of interindividual difference measures when private solutions were not attainable ($c_i = \infty$).

**coefficient**	**estimate**	**p**
Intercept	38.31	0.022
social value orientation	0.20	0.076
risk aversion	-0.26	0.970
age	0.79	0.226
sex (0 = female, 1 = male)	1.07	0.714
		**std. dev.**
$\sigma_{level\ 1}$		7.70
$\sigma_{level\ 2}$		5.20

Supplementary Table 13. Interindividual differences and cooperation (e =120).

Average cooperation (RP invested towards a public solution) as a function of interindividual difference measures when private solutions were attainable ($c_i \neq \infty$).

coefficient	estimate	p
Intercept	17.02	0.539
social value orientation	0.69	0.001
risk aversion	0.81	0.942
age	0.19	0.859
sex (0 = female, 1 = male)	2.29	0.642
		**std. dev.**
$\sigma_{\text{level 1}}$		11.00
$\sigma_{\text{level 2}}$		13.00

For participants with *e* = 60, social preferences were not significantly correlated with cooperation, neither when they relied on public solutions (Supplementary Table 14, see also Supplementary Figure 18) nor when they could afford to solve the problem independent of the group (Supplementary Table 15, see also Supplementary Figure 18).

Importantly, age or sex did not predict the extent of cooperation in any of the models. Risk preferences were only related to cooperation rates for participants with *e* = 60 when they had attainable private solutions (Supplementary Table 15).

Supplementary Table 14. Interindividual differences and cooperation (e = 60).

Average cooperation (RP invested towards a public solution) as a function of interindividual difference measures when private solutions were not attainable for the decision maker.

coefficient	estimate	p
Intercept	30.00	0.000
social value orientation	0.00	0.995
risk aversion	13.56	0.062
age	-0.33	0.195
sex (0 = female, 1 = male)	2.91	0.272
		**std. dev.**
$\sigma_{level\ 1}$		6.20
$\sigma_{level\ 2}$		5.00

Supplementary Table 15. Interindividual differences and cooperation (e =60).

Average cooperation (RP invested towards a public solution) as a function of interindividual difference measures when private solutions were attainable for the decision maker.

coefficient	estimate	p
Intercept	8.94	0.206
social value orientation	0.12	0.202
risk aversion	16.95	0.041
age	-0.17	0.532
sex (0 = female, 1 = male)	1.62	0.586
		**std. dev.**
$\sigma_{level\ 1}$		6.20
$\sigma_{level\ 2}$		8.60

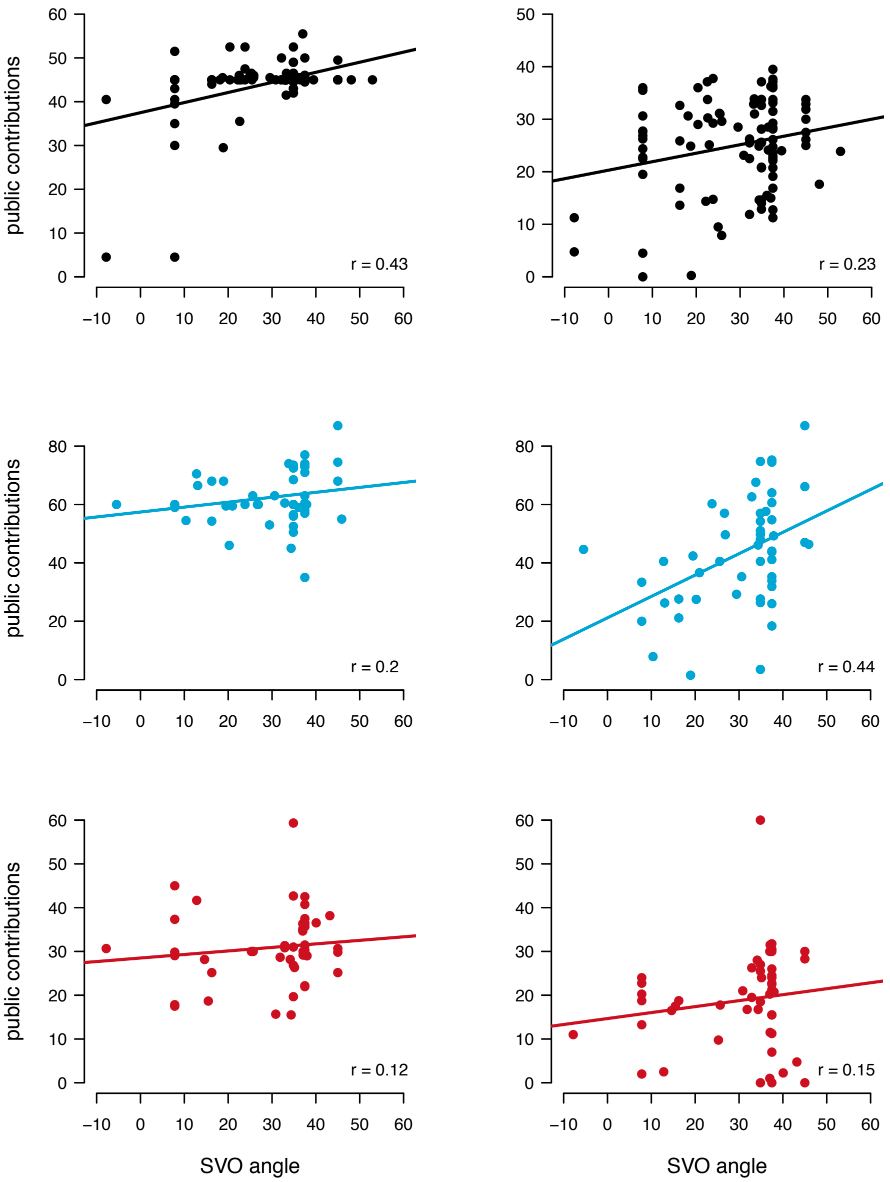

Supplementary Figure 18. Social Preferences and cooperation. Association between average public contributions and social value orientation angle (i.e. social preference, based on the social value orientation slider measure) for participants with $e = 90$ (upper panels, black), $e = 120$ (middle panels, blue), and $e = 60$ (lower panels, red) when private solutions were not available (left panels) vs. available (right panels). Each dot indicates one participant. Lines indicate best linear fit based on least-squares.

Supplementary References

1. Henrich, J. *The secret of our success: How culture is driving human evolution, domesticating our species, and making us smarter.* (Princeton University Press, 2015).

2. Tomasello, M., Melis, A. P., Tennie, C., Wyman, E. & Herrmann, E. Two key steps in the evolution of human cooperation. *Curr Anthropol* **53,** 673–692 (2012).

3. Roberts, G. Cooperation through interdependence. *Anim Behav* **70,** 901–908 (2005).

4. Lindenberg, S. Sharing groups: Theory and suggested applications. *J Math Sociol* **9,** 33–62 (1982).

5. Lindenberg, S. in *Soziale Bedingungen - Individuelles Handeln - Soziale Konsequenzen* 1–19 (1985).

6. Lindenberg, S. in *Paradoxical effects of social behavior* (eds. Diekmann, A. & Mitter, P.) 297–310 (Physica-Verlag HD, 1986).

7. Hacker, J. S. Privatizing risk without privatizing the welfare state: The hidden politics of social policy retrenchment in the United States. *Am Polit Sci Rev* **98,** 243–260 (2004).

8. Milinski, M., Hilbe, C., Semmann, D., Sommerfeld, R. & Marotzke, J. Humans choose representatives who enforce cooperation in social dilemmas through extortion. *Nat Comm* **7,** 10915 (2016).

9. Milinski, M., Sommerfeld, R. D., Krambeck, H.-J., Reed, F. A. & Marotzke, J. The collective-risk social dilemma and the prevention of simulated dangerous climate change. *Proc Natl Acad Sci USA* **105,** 2291–2294 (2008).

10. Milinski, M., Semmann, D., Krambeck, H.-J. & Marotzke, J. Stabilizing the Earth's climate is not a losing game: Supporting evidence from public goods experiments. *Proc Natl Acad Sci USA* **103,** 3994–3998 (2006).

11. Tavoni, A., Dannenberg, A., Kallis, G. & Löschel, A. Inequality, communication, and the avoidance of disastrous climate change in a public goods game. *Proc Natl Acad Sci USA* **108,** 11825–11829 (2011).

12. Gradstein, M. & Nitzan, S. Binary participation and incremental provision of public goods. *Soc Choice Welf* **7,** 171–192 (1990).

13. Murphy, R. O., Ackermann, K. A. & Handgraaf, M. Measuring social value orientation. *Judgm Decis Mak* **6,** 771–781 (2011).

14. Falk, A., Becker, A., Dohmen, T., Huffman, D. & Sunde, U. The Preference Survey Module: A Validated Instrument for Measuring Risk, Time, and Social Preferences. *working paper* 1–69 (2016). DOI: 10.2139/ssrn.2725874

15. Kuznetsova, A., Brockhoff, P. B. & Christensen, R. H. B. Lmertest package: Tests in linear mixed effects models. *J Stat Softw* **82,** 1–26 (2017).

16. van Dijk, E. & Wilke, H. Differential interests, equity, and public good provision. *J Exp Soc Psychol* **29,** 1–16 (1993).

17. Spiller, J., Ufert, A., Vetter, P. & Will, U. Norms in an asymmetric public good experiment. *Econ Lett* **142,** 35–44 (2016).

18. Gross, J. & De Dreu, C. K. W. Individual solutions to shared problems create a modern tragedy of the commons. *Science Advances* **5,** 1–8 (2019).
