# Supplementary material for: Self-reliance crowds out group cooperation and increases wealth inequality
Source: Nat Commun. 2020 Oct 14;11:5161. doi: 10.1038/s41467-020-18896-6 (PMC7560835; doi:10.1038/s41467-020-18896-6)
Supplement: Supplementary file 2 — Reporting Summary [file 41467_2020_18896_MOESM2_ESM.pdf]

nature research

Corresponding author(s): Jörg Gross

Last updated by author(s): Aug 12, 2020

Reporting Summary

Nature Research wishes to improve the reproducibility of the work that we publish. This form provides structure for consistency and transparency in reporting. For further information on Nature Research policies, see our Editorial Policies and the Editorial Policy Checklist.

Statistics

For all statistical analyses, confirm that the following items are present in the figure legend, table legend, main text, or Methods section.

n/a	Confirmed	
[ ]	[x]	The exact sample size (*n*) for each experimental group/condition, given as a discrete number and unit of measurement
[x]	[ ]	A statement on whether measurements were taken from distinct samples or whether the same sample was measured repeatedly
[ ]	[x]	The statistical test(s) used AND whether they are one- or two-sided<br>*Only common tests should be described solely by name; describe more complex techniques in the Methods section.*
[ ]	[x]	A description of all covariates tested
[ ]	[x]	A description of any assumptions or corrections, such as tests of normality and adjustment for multiple comparisons
[ ]	[x]	A full description of the statistical parameters including central tendency (e.g. means) or other basic estimates (e.g. regression coefficient) AND variation (e.g. standard deviation) or associated estimates of uncertainty (e.g. confidence intervals)
[ ]	[x]	For null hypothesis testing, the test statistic (e.g. *F*, *t*, *r*) with confidence intervals, effect sizes, degrees of freedom and *P* value noted<br>*Give P values as exact values whenever suitable.*
[x]	[ ]	For Bayesian analysis, information on the choice of priors and Markov chain Monte Carlo settings
[ ]	[x]	For hierarchical and complex designs, identification of the appropriate level for tests and full reporting of outcomes
[x]	[ ]	Estimates of effect sizes (e.g. Cohen's *d*, Pearson's *r*), indicating how they were calculated

Our web collection on statistics for biologists contains articles on many of the points above.

Software and code

Policy information about availability of computer code

Data collection	No software was used. Experiments were programmed in HTML, PHP, and JavaScript by the first author.
Data analysis	Data analyses were performed in R (version 3.3.3)

For manuscripts utilizing custom algorithms or software that are central to the research but not yet described in published literature, software must be made available to editors and reviewers. We strongly encourage code deposition in a community repository (e.g. GitHub). See the Nature Research guidelines for submitting code & software for further information.

Data

Policy information about availability of data

All manuscripts must include a data availability statement. This statement should provide the following information, where applicable:

- Accession codes, unique identifiers, or web links for publicly available datasets
- A list of figures that have associated raw data
- A description of any restrictions on data availability

Raw data for all experiments are publicly available in an Open Science Framework (OSF) repository (https://osf.io/twv7b/?view_only=0bfe1e89484d4bc6a727a9e1a9073844). Source data are provided with this paper.

Field-specific reporting

Behavioural & social sciences study design

All studies must disclose on these points even when the disclosure is negative.

Study description	quantitative study, lab experiment using an economic game to investigate group cooperation when groups are faced with a shared problem that can be also solved individually.
Research sample	Leiden University undergraduates (mean age = 22.5, sd = 3.5, 146 female). Sample is not representative of the dutch population but provides insights into a general causal mechanism according to the standards of the discipline.
Sampling strategy	Participants were invited from an existing database of volunteers (ORSEE). Participants were randomly allocated to treatments. Sample size was determined based on previous studies and standards in the field (see Thielmann, I., Spadaro, G., & Balliet, D. (2020). Personality and Prosocial Behavior: A Theoretical Framework and Meta-Analysis. Psychological Bulletin, 146(1), 30–90 and Zelmer, J. (2003). Linear Public Goods Experiments: A Meta-Analysis. Experimental Economics, 6(3), 299–310).
Data collection	Participants were assigned to individual cubicles where they performed the experiment in front of a computer. Sessions were conducted by two experimenters. Experimenters were blind to the treatment conditions conducted and were only present to answer comprehension questions and for the payment procedure.
Timing	Data collection was performed without gaps between February and March 2019.
Data exclusions	No data were excluded.
Non-participation	No participants dropped out of the experiment.
Randomization	Participants were randomly allocated to treatment conditions.

Reporting for specific materials, systems and methods

We require information from authors about some types of materials, experimental systems and methods used in many studies. Here, indicate whether each material, system or method listed is relevant to your study. If you are not sure if a list item applies to your research, read the appropriate section before selecting a response.

Materials & experimental systems

n/a	Involved in the study	
☒	☐	Antibodies
☒	☐	Eukaryotic cell lines
☒	☐	Palaeontology and archaeology
☒	☐	Animals and other organisms
☐	☒	Human research participants
☒	☐	Clinical data
☒	☐	Dual use research of concern

Methods

n/a	Involved in the study	
☒	☐	ChIP-seq
☒	☐	Flow cytometry
☒	☐	MRI-based neuroimaging

Human research participants

Policy information about studies involving human research participants

Population characteristics	See above.
Recruitment	Students were recruited through the online recruitment platform of our lab at Leiden University. Recruitment e-mails invited participants to a study on "decision making" without providing any further details on the nature of the study to avoid self-selection.
Ethics oversight	Psychology Research Ethics Committee, Department of Psychology, Leiden University.

Note that full information on the approval of the study protocol must also be provided in the manuscript.
